# Ecological speciation by temporal isolation in a population of the stonefly *Leuctra hippopus* (Plecoptera, Leuctridae)

**DOI:** 10.1002/ece3.2638

**Published:** 2017-02-10

**Authors:** Louis Boumans, Silje Hogner, John Brittain, Arild Johnsen

**Affiliations:** ^1^ Natural History MuseumUniversity of OsloOsloNorway

**Keywords:** AFLP, cytochrome c oxidase subunit I, freshwater insects, genetic differentiation, incipient speciation, RAD‐based SNPs

## Abstract

Stream dwelling invertebrates are ideal candidates for the study of ecological speciation as they are often adapted to particular environmental conditions within a stream and inhabit only certain reaches of a drainage basin, separated by unsuitable habitat. We studied an atypical population of the stonefly *Leuctra hippopus* at a site in central Norway, the Isterfoss rapids, in relation to three nearby and two remote conspecific populations. Adults of this population emerge about a month earlier than those of nearby populations, live on large boulders emerging from the rapids, and are short‐lived. This population also has distinct morphological features and was studied earlier during the period 1975–1990. We reassessed morphological distinctness with new measurements and added several analyses of genetic distinctness based on mitochondrial and nuclear sequence markers, as well as AFLP fingerprinting and SNPs mined from RAD sequences. The Isterfoss population is shown to be most closely related to its geographical neighbors, yet clearly morphologically and genetically distinct and homogeneous. We conclude that this population is in the process of sympatric speciation, with temporal isolation being the most important direct barrier to gene flow. The shift in reproductive season results from the particular temperature and water level regime in the Isterfoss rapids. The distinct adult body shape and loss of flight are hypothesized to be an adaptation to the unusual habitat. Ecological diversification on small spatial and temporal scales is one of the likely causes of the high diversity of aquatic insects.

## 1 Introduction

The adaptation of local populations to different environments can lead to spatial isolation and temporal isolation, both common factors in ecological speciation (Coyne & Orr, 2004: 179 ff.; Rundle & Nosil, 2005). Temporal isolation, which occurs when sympatric populations have nonoverlapping reproductive periods, has not been as thoroughly investigated as other isolating barriers (Coyne & Orr, 2004:204). Most examples concern plant populations that differ in flowering times (cited in Lowry, Modliszewski, Wright, Wu, & Willis, 2008) and allochronically spawning populations of fish and corals (e.g., Barson, Haugen, Vøllestad, & Primmer, 2009; Limborg, Waples, Seeb, & Seeb, 2014; Villanueva, 2016). In insects, periodic cicadas constitute a famous case (Marshall & Cooley, 2000), while Sota, Kagata, Ando, Utsumi, and Osono (2014) report incipient speciation in early and late winter populations of winter moths. Freshwater insects with synchronized adult emergence and a short adult life stage, particularly Ephemeroptera and Plecoptera, are expected to be good systems to study diversification by temporal isolation, but so far studies have been few and inconclusive (Dijkstra, Monaghan, & Pauls, 2014). Here, we present evidence of temporal isolation in a stream dwelling stonefly.

Stream dwelling invertebrates face habitat fragmentation on a variety of spatial scales. Their linear habitats generally cover less area and support smaller populations than most terrestrial or marine habitats (Whittaker & Likens, 1973), yet they harbor a relatively large proportion of all known animal species, particularly insects (Dijkstra et al., 2014; Dudgeon et al., 2006). Running waters (lotic habitat) are geologically more stable than still waters (lentic habitat), which may explain why lentic insects tend to be more capable of dispersion, while lotic insects demonstrate more genetic differentiation (Hof, Brändle, & Brandl, 2006; reviewed in Dijkstra et al., 2014). Genetic differentiation between drainage basins has been reported for many aquatic animals (e.g., Monaghan, Spaak, Robinson, & Ward, 2002; Phillipsen & Metcalf, 2009; Schmidt, Bart, Nyingi, & Gichuki, 2014) and is most pronounced in flightless species restricted to headwater habitats (Hughes, 2007). Within a drainage basin, drift leads to a predominantly downstream migration, while steep waterfalls constitute barriers to upstream dispersal for nonflying animals. The fauna of individual headwaters is therefore often more isolated and genetically less diverse than in higher‐order streams (Alp, Keller, Westram, & Robinson, 2012; Wofford, Gresswell, & Banks, 2005), while the diversity among headwaters is high (Finn, Bonada, Múrria, & Hughes, 2011). Moving from headwaters to higher‐order streams, habitat characteristics like aquatic and riparian vegetation, stream velocity and bottom sediment change, and organisms are typically adapted to particular parts of the drainage basin (McCabe, 2010).

In general, winged aquatic insects are capable of dispersal over a longer distance (c. 0.5–1 km) in the adult stage, but the majority of specimens do not fly far from their emergence site and generally follow the watercourse (e.g., Briers, Cariss, & Gee, 2002; Briers, Gee, Cariss, & Geoghegan, 2004; Griffith, Barrows, & Perry, 1998; Macneale, Peckarsky, & Likens, 2005; Petersen, Masters, Hildrew, & Ormerod, 2004). While incidental longer‐range dispersal is important for the (re)colonization of new habitat patches, the limited gene flow resulting from short average dispersal allows for genetic adaptations to the local environment driven by differential selective pressure. In aquatic invertebrates, intraspecific genetic divergence is often found on small geographical scales (<50 km) (e.g., Alp et al., 2012; Yaegashi, Watanabe, Monaghan, & Omura, 2014). Watanabe, Kazama, Omura, and Monaghan (2014) found that divergence in three caddisflies and one mayfly at genetic loci under selection correlated most strongly with the ecological factor that was most limiting to the species' distribution (elevation, concentrations of algae, or ammonia nitrogen). Laboratory studies of stoneflies have identified a number of inheritable population‐specific life cycle characteristics, including the occurrence of egg diapause (Khoo, 1968), duration of nymphal growth period (Lillehammer, 1975b), and optimal temperatures for egg development and nymphal growth (Brittain, Lillehammer, & Saltveit, 1986; Lillehammer, 1987; Lillehammer, Brittain, Saltveit, & Nielsen, 1989).

While genetic divergence of populations in separate drainage basins may lead to allopatric speciation by genetic drift, divergence of populations adapting to contrasting environments within a drainage basin may lead to reproductive isolation, a process known as ecological speciation (Rundle & Nosil, 2005; Schluter, 2009). Assortative mating, immigrant inviability, and reduced fitness of hybrids enhance ecological speciation (Schluter, 2009). As an example of ecological selection, Yaegashi et al. (2014) found populations of the caddisfly *Stenopsyche marmorata* to be structured into upstream and downstream clusters, rather than according to catchments. Longitudinal structuring of populations within a drainage basin may be enhanced by lakes that constitute a barrier to gene flow in lotic species (Monaghan, Spaak, Robinson, & Ward, 2001). Such structuring may be an early stage of parapatric ecological speciation and is in line with the observation that congeneric aquatic insect species often inhabit overlapping reaches of a drainage basin (e.g., Kaćanski, 1971; Rupprecht, 1983; Gordon, Wallace, & Grubaugh, 1998; Statzner & Dolédec, 2011; Boumans & Murányi, 2014; reviewed in Dijkstra et al., 2014).

Our study addresses a case where adaptation to a small stretch of running water necessitated phenological adaptation, leading to temporal isolation and putative or incipient speciation. The widespread, spring‐emerging West Palearctic stonefly, *Leuctra hippopus* Kempny 1899, has a morphologically and phenologically atypical population in the Isterfoss rapids in central Norway, previously described and studied by Lillehammer (1974, 1975b, 1976, 1986, 1987) and Ørmen (1991). Our main hypothesis is that this population has become genetically isolated from conspecifics in nearby streams due to earlier emergence and short adult life, with the lack of gene flow enhancing genetic adaptation to local conditions. An alternative hypothesis is that the Isterfoss population has an historical origin that is different from other Scandinavian populations (Ørmen, 1991: 63–64). Following the retreat of ice sheets at the start of the Holocene, about 10,000 years B.P. (Mangerud, Gyllencreutz, Lohne, & Svendsen, 2011), stoneflies, like several other taxa, have populated the Scandinavian Peninsula from the south, northeast, or both (Boumans & Baumann, 2012; Boumans & Brittain, 2012; Lillehammer, 1988: 25–27).

In order to test the temporal isolation hypothesis, we reassessed the morphological distinctness of the *L. hippopus* population from Isterfoss with additional morphometric measurements. Subsequently, we analyzed mitochondrial and nuclear sequence data in order to assess the phylogenetic position of the Isterfoss population relative to Scandinavian and Western European conspecifics, testing the hypothesis of a distinct origin. Thirdly, we used genomewide sampled molecular markers (AFLP and SNPs) to establish the degree of genetic distinction and admixture of *L. hippopus* from Isterfoss and five additional sites at distances ranging from 8 to 1,280 km. With these analyses, we tested the hypothesis that Isterfoss is genetically homogeneous and distinct from its geographical neighbors. If the hypothesis of a distinct historical origin can be refuted, a low degree of admixture is considered indicative of (ongoing) local diversification and incipient speciation.

## 2 Materials and Methods

### 2.1 Study species and locality

**Figure 1:**
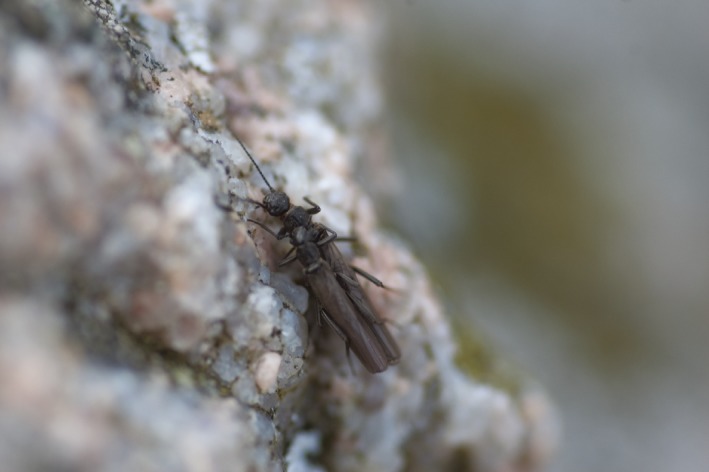
*Leuctra hippopus* Kempny, mating pair on one of the boulders in the Isterfoss rapids. Photograph taken by L. Boumans on 23 April 2011

*Leuctra hippopus* (Plecoptera, Leuctridae; Figure 1) is a detritivore stonefly that is widely distributed in streams of the western Palaearctic (Graf et al., 2009). It has a 1‐year life cycle, with a short‐lived adult stage. The time of emergence in Fennoscandia varies from early March in coastal areas with oceanic climate, to June and July at high altitude and latitudes (Lillehammer, 1988: 148). Adults of typical populations live in the riparian zone and are capable of flight. A high degree of morphological variation in both males and females of *L. hippopus* has been documented for specimens collected from different localities in Norway, notably in the shape of abdominal tergites as well as wing length and wing venation (Lillehammer, 1974, 1976, 1985, 1986; Ørmen, 1991: 36–41). While there is also within‐site morphological variation, the species has a tendency to produce local forms. The most characteristic and best documented local form occurs in the Isterfoss rapids.

While all closely studied Norwegian populations of *L. hippopus* show within‐population variation in the shape of the abdominal sclerites, the population in Isterfoss is relatively homogeneous (Lillehammer, 1974, 1976). Likewise, Isterfoss is the only Norwegian population that has been reported to be homogeneously short‐winged (Lillehammer, 1974, 1976). It is also relatively homogeneous in terms of variation in wing venation patterns, with a high proportion of individuals with an irregular pattern that occurs only at lower frequencies elsewhere (Lillehammer, 1974). The irregular wing venation is a heritable trait that was maintained in stoneflies reared in laboratory for two generations (Lillehammer, 1976, 1986). Unlike typical *L. hippopus*, the specimens from Isterfoss do not fly (Ørmen, 1991: 13; and own observations). Finally, they are more pigmented (Lillehammer, 1976) and more stockily built (Ørmen, 1991: 13) than other populations.

Isterfoss is a 130‐m‐long and 60‐m‐broad rapids at 650 m a.s.l. connecting the lakes Isteren and Galthåen in the Norwegian county of Hedmark. The central part is a deep, fast running stream with a bottom of large stones (20–50 cm diameter) and boulders (50–200 cm) of moraine origin, flanked on both sides by a broad floodplain. The central stream and the floodplains are separated by two rows of large boulders that rise above the water surface and are covered with lichens and mosses (illustrations of the study site are included in Figures S1–S3). Adults of *L. hippopus* occur only on these central boulders where they aggregate, feed, and mate (Figures 1 and S4). No specimens were found on boulders on the floodplains, nor in the vegetation on the shores, nor on boulders without lichen cover. Neither we nor previous researchers ever collected *L. hippopus* nymphs by means of kick sampling in the floodplains, despite repeated attempts and despite the occurrence of other benthic fauna (Ørmen, 1991: 13). Also because the population is flightless, we assume that the eggs and nymphs develop in the hyporheic zone among and underneath the same boulders where the adults were collected. On the stream side of these boulder rows, stream velocity is high (1 m/s, measured on 8 May 2012, 50 cm from the edge, at 50 cm depth), and any eggs deposited there would be flushed into Lake Galthåen and be lost as lakes are unsuitable habitat for this species. On the outer, floodplain side of the boulder rows and between boulders in a row, stream velocity is low (0–0.25 m/s on the same date).

**Figure 2:**
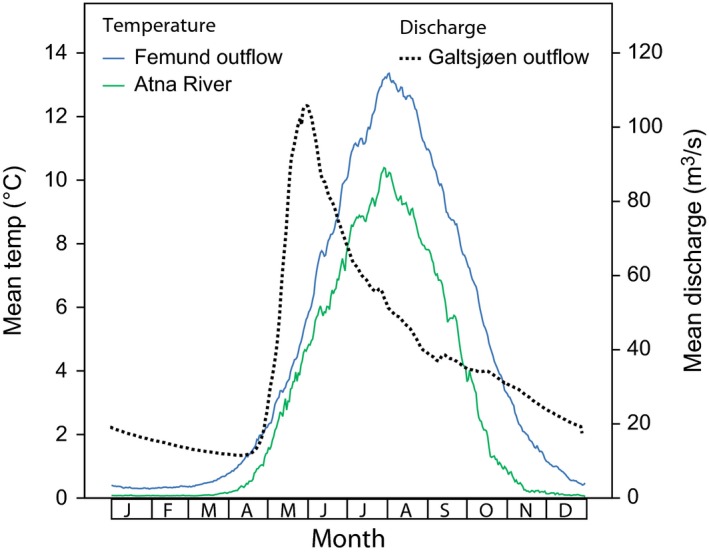
Habitat characteristics at Isterfoss rapids. Solid lines: Mean water temperature in the outflow of Lake Femund (61°56.094′N 11°52.034′E) measured daily in the period 14 August 1984–20 August 2013 (higher values), and in the Atna River (62°00.604′N 9°59.986′E), measured 02 June 1987–21 May 2014. The outflow of Lake Femund is c. 5.3 km upstream from the outflow of Lake Isteren at Isterfoss, and the temperatures at both sites are assumed to be very similar. Dotted line: Mean daily discharge from Isterfoss measured at Lake Galtsjøen (61°53.479′N 11°45.227′E) in the period 26 June 1949–31 December 1983. Data source: Norwegian Water Resources and Energy Directorate

This part of central Norway has a continental and dry climate, with meteorological winter lasting from the middle of October to the middle of April (Norwegian Meteorological Institute s.a.). However, the continuous strong current and the slightly warmer waters from Lake Isteren keep the central part of Isterfoss open throughout the year and lead to a later cooling in autumn and an early break‐up of the ice cover in the spring (Lillehammer, 1975b; Ørmen, 1991: 12). We obtained long‐term, year‐round measurements of temperature from nearby measurement stations from the Norwegian Water Resources and Energy Directorate (NVE). The temperature of the outflow of Lake Isteren at Isterfoss can be assumed to be similar to that measured at the outflow of Lake Femund, c. 5.3 km upstream from Isterfoss (Figure S1). (In this lake outflow*, L. hippopus* does not occur.) Temperature data were also available from the Atna River, c. 5 km upstream from our additional sample collecting site on the Atna River (Folldal, see below). The temperature regimes of these two sites are typical for Norwegian lake outflows and mountain rivers, respectively (Asvall, 1994). The temperature is consistently higher in the lake outflow than in the river, the difference ranging from about 0.3°C in winter to 3.3°C in August and September (Figure 2). Changes in water level at Isterfoss can be deduced from the daily discharge data collected downstream from Isterfoss at Lake Galtsjøen. Sudden water level rise resulting from snowmelt occurs in the first 3 weeks of May (Figure 2).

In Isterfoss, adult *L. hippopus* are typically present during the second half of April and the first half of May (Lillehammer, 1975a, 1987), although emergence is earlier in some years (Ørmen, 1991: 11; Cleven, s.a. 1992: 17). At the time of emergence at Isterfoss, the surrounding landscape is still partly snow‐covered (own observation), and in some years, other streams are still covered with ice (Ørmen, 1991: 14). Emergence in Isterfoss is much earlier than in nearby streams (Lillehammer, 1987). For instance, in a stream in Tynset, 65 km away and 500 m a.s.l., adult *L. hippopus* are present in June (Lillehammer, 1975a). Specimens from the nearby stream Hølbekken (see below) also emerge about 1 month later than in Isterfoss (own observations). Laboratory experiments have shown that nymphal growth and timing of adult emergence of *L. hippopus* are determined by water temperature as well as population‐specific genetic factors (Lillehammer, 1975b, 1987). The life span of adults from Isterfoss is only about a week, compared to 4 weeks or more for other Norwegian populations. As a consequence, sexual maturity is reached sooner after emergence, and females lay fewer eggs (Lillehammer, 1975b; Ørmen, 1991: 13–14, 22). The relatively high temperature of the lake outflow (Figure 2) enables faster nymphal growth and an earlier emergence.

### 2.2 Specimens

In order to establish the monophyly of the species and the phylogenetic position of the Isterfoss population in Scandinavia and Western Europe, we sequenced the mitochondrial marker cytochrome c oxidase subunit I (COI) from 55 *L. hippopus* specimens collected in northern, central, and southern Europe, either collected by ourselves or donated by colleagues. In addition, we included 12 specimens in total of the following closely related species as outgroup: *L. andalusiaca* Aubert 1962, *L. elisabethae* Ravizza 1985, *L. hippopoides* Kacanski & Zwick 1970, and *L. pseudohippopus* Rauser 1965.

The nuclear markers 28S and ITS were sequenced for a subset of these. Specimens used in molecular research are preserved in 96% ethanol; all specimens are deposited in the DNA bank of the Natural History Museum, University of Oslo (NHMO). An overview of the specimens is presented in Table S1.

**Figure 3:**
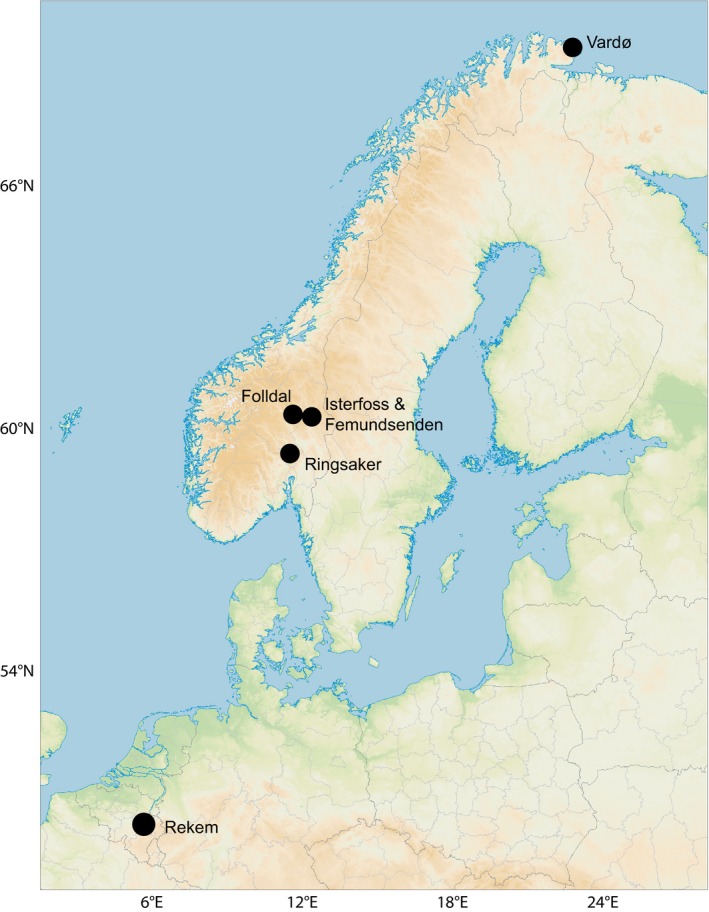
The six sampling sites for morphometric measurements, AFLP and RAD tag sequencing in Norway and Belgium. Sites Isterfoss and Femundsenden cannot be distinguished at this scale

For the characterization of the Isterfoss population by means of morphometry, AFLP fingerprinting, and SNP analysis, we used adult specimens collected in Isterfoss on 23 April 2011 and 8 May 2012. These were analyzed together with specimens from five additional collecting sites (Figure 3, Table 1). We refer to the collecting sites in Table 1 as statistical “populations” in the context of AFLP and SNP sequence analysis, while they are not necessarily, or not all to the same degree, distinct populations in the biological sense.

**Table 1:** Sites where *Leuctra hippopus* specimens were collected for analysis of AFLP, RAD, and morphological characters. Distance = arial distance from Isterfoss, m = male, f = female. The Ringsaker population was not included in the AFLP analysis

Site	Province	Country	Coordinates	Altitude m asl	Distance	Drainage basin	N morph.	N AFLP	N RAD
Isterfoss rapids	Hedmark	Norway	61°54.599′N 11°46.698′E	648	0	Trysil	10 m 16f	18	5
Femundsenden: Hølbekken stream (Figure S5)	Hedmark	Norway	61°55.146′N 11°55.674′E	666	8 km NE	Trysil	2 m 3f	5	5
Folldal: Upper Atna River	Hedmark	Norway	61°59.053′N 10°01.771′E	717	92 km NW	Glomma	4 m 4f	9	5
Ringsaker: Badhusbekken stream	Hedmark	Norway	60°58.949′N 10°38.518′E	124	120 km SW	Glomma	1 m 4f	0	4
Vardø: Næringselva stream	Finnmark	Norway	70°26.281′N 0°52.921′E	16	1272 km NE	Coastal	10 m 10f	12	3
Rekem: Ziepbeek stream	Limburg	Belgium	50°55.467′N 5°39.241′E	52	1280 km SW	Meuse	3 m 1f	4	3

### 2.3 Morphometry

Morphometrical measurements were used to reassess the morphological distinctness of the Isterfoss population. Lillehammer (1974) measured the wing length relative to the whole body length. However, stoneflies are soft‐bodied animals that are stretched to varying degrees as a result of egg production, desiccation, or postcollection treatment, which complicates the measurement of body length. For this reason, we reassessed wing length variation, this time dividing the wing length by the width of the head as a proxy for body size (Beer‐Stiller & Zwick, 1995; Zwick, 1992). We measured specimens from Isterfoss and the additional sites (Table 1) using a Dino‐Lite^®^ eyepiece camera and the DinoCapture 2.0 software. Wing length is the average of left and right forewing; head width was likewise averaged over two measurements taken across the eyes in dorsal view (i.e., the distance between the left border of the left eye and the right border of the right eye).

Table 1 lists the number of samples per sex and per site. For statistical analysis, the samples from Ringsaker, Femundsenden, and Folldal were pooled into one population (“other Hedmark”) in order to create three populations of similar sample size, that is, Isterfoss, other Hedmark, and Vardø. Genomewide sampled DNA markers provide justification for grouping these three other Hedmark collecting sites (see Results section). A one‐way ANOVA with Tukey's post hoc test, performed in SPSS v.22, was used for a statistical evaluation of the pairwise difference in morphology between the following three Norwegian populations: Isterfoss, the pooled specimens of the three other collecting sites in the Hedmark county (“other Hedmark”), and Vardø. Measurements of the Belgian samples are provided in Table S2 but not analyzed statistically due to the low number of samples. The ANOVA was run separately for female and male specimens.

### 2.4 Sequence data

We extracted DNA from the head plus prothorax or, in the case of samples used for restriction site‐associated DNA (RAD) sequencing, the entire body except the abdomen. The GeneMole extraction robot was used for all extractions used in AFLP or RAD tag protocols; for specimens used for sequence data, we also used the Qiagen DNeasy Blood and Tissue Kit. In both cases, we followed the manufacturers' protocols. Skeleton parts were not crushed but retrieved after DNA extraction and stored with the remainder of the specimen.

The mitochondrial marker COI of *L. hippopus* specimens and outgroup taxa was amplified with the protocol described in Boumans and Baumann (2012). Part of the COI sequences was produced at the sequencing facility of the Canadian Centre for DNA Barcoding in Guelph in the framework of the DNA barcoding projects NorBol—Freshwater Insects (NOEPT) and West Palaearctic Plecoptera (WPPLE), and retrieved from the Barcode of Life Data System (BOLD) (cf. Ratnasingham & Hebert, 2007). Two *L. hippopus* sequences, labeled HM376116 and BI2019, from, respectively, the Barcoding Fauna Bavarica project (Hendrich et al., 2010) and NTNU Student projects in DNA barcoding (STUBA) were likewise downloaded from BOLD and added to the data matrix. In order to assess whether mitochondrial haploclades shared between Isterfoss and other Norwegian populations could result from introgression (Boumans & Tierno de Figueroa, 2016), we sequenced nuclear markers in a subset of *L. hippopus* and outgroup taxa. The D1–D2 region of the large‐subunit (28S) rRNA gene was amplified and sequenced with the primers and methods described in Boumans and Baumann (2012). Finally, the ITS region covering ITS1, 5.8S, and ITS2 and adjacent fragments of 18S and 28S was amplified and sequenced for a subset of *L. hippopus* and *L. elisabethae* specimens with primers ITS‐1‐SP18 and ITS‐1‐SP28 (McLain, Wesson, Oliver, & Collins 1995) following the methods described in Boumans and Murányi (2014). GenBank accession numbers of all sequences used are given in Table S1.

The package Geneious version 6.0.5 was used for sequence editing and alignment. Phylogenetic trees of the COI sequences were constructed with distance (neighbor joining, NJ) and maximum‐parsimony (MP) methods implemented in Paup*, and with Bayesian inference (BI) in MrBayes version 3.2 (Ronquist & Huelsenbeck, 2003). Details are given in Appendix S1. The 28S and ITS data matrices were not suitable for phylogenetic inference due to, respectively, lack of sequence variation and variation occurring largely in indels. The most divergent ITS sequences are separated by two 3‐ to 5‐bp‐long indels whose homology and alignment is doubtful. However, to visualize the sequence divergence in ITS, we created a single most parsimonious tree based on an exhaustive tree search in which “gaps” were treated as a fifth character state.

### 2.5 AFLP fingerprinting

We performed AFLP fingerprinting of 56 specimens, representing the collecting sites at Isterfoss, Femundsenden, Folldal, and Vardø in Norway and Rekem in Belgium (Table 1). (Samples from the Ringsaker site were not included in this analysis.) The AFLP laboratory protocol and data collecting procedure followed Alsos et al. (2007) and Skrede, Borgen, and Brochmann (2009) with minor modifications. As scoring by means of visual inspection introduces a high degree of subjectivity (Bonin et al., 2004), we analyzed unedited peak heights in the size range from 50 to 500 bp output by GeneMapper with the R program AFLPScore version 1.4 (Whitlock, Hipperson, Mannarelli, Butlin, & Burke, 2008). After removing badly performing samples and markers, we retained 109 markers in 48 specimens representing the five above‐mentioned populations. The average number of markers scored as present per specimen was 19.7 ± 4.5 (range 9–28). Error rates were calculated with the help of 13 replicated samples and ranged from 2.8% to 3.2% for three subsets produced with different selective primer pairs. Details of the laboratory and peak scoring protocols are given in Appendix S1.

We used Bayesian approaches implemented in the software packages Structure version 2.3.3 (Falush, Stephens, & Pritchard, 2007; Pritchard, Stephens, & Donnelly, 2000) and Bayesian Analysis of Population Structure (BAPS) (Corander, Marttinen, Siren, Tang, & Cheng, 2005; Corander, Waldmann, & Sillanpää, 2003) to infer genetic population structure on the basis of the absence–presence data. The input files for these programs were created with the R program AFLPDat (Ehrich, 2006). Structure was used to infer the most likely clustering of sample specimens into population clusters. We allowed for individuals to have mixed ancestry (admixture model) and did not use the sampling sites as prior. The maximum number of inferred clusters was set to 7, allowing for the recognition of each sampling site as a cluster, in addition to some degree of site internal clustering. For each value of *K* in the range 1–7, 10 runs were performed each consisting of a burn‐in phase of 500,000 repetitions followed by 1 million repetitions. Remaining settings were default values. Structure outputs a cluster membership coefficient matrix showing each individual's estimated proportion of membership in each of the *K* clusters, and visualizes this in a histogram. The average and standard deviation was calculated for the ten estimated Ln Prob of Data for each value of *K*. The optimal number of clusters was identified by the measure ΔK (Evanno, Regnaut, & Goudet, 2005), which weighs the change in posterior probability for increasing number of clusters by the standard deviation among runs. The web application Structure Harvester (Earl & Vonholdt, 2012) was used to calculate and graphically display Δ*K* for *K* = 2 to *K* = 7. For each number of *K*, the estimated cluster membership coefficient matrices of the ten runs of Structure were fed into CLUMPP version 1.1.2 (Jakobsson & Rosenberg, 2007) to produce a mean matrix and corresponding histogram. The software Distruct (Rosenberg, 2004) and a graphics application were used for the editing of the histogram.

For comparison, we also analyzed the AFLP data matrix in BAPS with the modules “Clustering of individuals,” “Admixture based on mixture clustering,”“Clustering of groups of individuals,” and “Spatial clustering of groups.” The resulting file of mixture clustering was used as input for the admixture analysis with the following settings: minimum size of population = 10, number of iterations for all individuals = 100, number of simulated reference individuals per population = 200, and number of iterations per reference individual = 10. When clustering groups of individuals, the five collecting sites were input as predefined groups, and decimal geographical coordinates were added for the spatial module. The maximum number of clusters was set as 2 and 20 so as to identify the most divergent specimens and find the optimal clustering, respectively. The module “Spatial clustering of groups” was used to produce a Voronoi tessellation graphic (Corander, Sirén, & Arjas, 2008).

### 2.6 Restriction site‐associated DNA (RAD) markers

We prepared RAD sequencing libraries for 25 stoneflies from six different populations using the restriction enzyme SfbI, following the protocol by Etter, Preston, Bassham, Cresko, and Johnson (2011) with some small modifications (details in Appendix S1). This RAD protocol produces libraries of genomic fragments that are determined at one end by the restriction enzyme cut site common to all individuals, and randomly shared the other end by means of sonication.

DNA fragments were ligated to a specimen‐specific barcode sequence, so that SNP data were obtained at the individual level. The library was sequenced together on one‐sixth of a single lane shared with another taxon on an Illumina Genome Analyzer II with a single read run, 100 bp long at the Norwegian Sequencing Center at Ullevål University Hospital in Oslo, Norway.

The generated SbfI library contained almost 76 million single‐end stonefly reads. However, the RAD tag library preparation turned out to have been inefficient, with many tags missing and an extraordinarily high duplication rate of the included tags (mean 95.2, range 95.0–95.5). We reduced the number of duplicates using the program clone_filter in Stacks v1.29. The output file of clone_filter was then used as input for the process_radtags program in Stacks (Catchen, Amores, Hohenlohe, Cresko, & Postlethwait, 2011).

After removing low‐quality and incomplete reads, we retained approximately 11 million reads, corresponding to c. 438,000 reads per specimen. There were large differences between specimens, with the number of reads ranging from 41,000 to 1,348,000. After removal of lower‐quality final sites, the reads were trimmed to a length of 86 nucleotides, including the first six invariable positions that belong to the restriction enzyme recognition site. Sufficient tags were obtained to allow for population genetics analysis, but measurements of heterozygosity may be biased. For this reason, we will not discuss population genetics measures based on heterozygosity, although we present these as supporting data (see Data accessibility).

We used the *denovo_map.pl* pipeline of programs in Stacks v1.29 (Catchen et al., 2011) to build loci de novo and detect haplotypes in each individual, build a catalog of loci, and match the individuals against this catalog. Filtering parameters in the tag library building phase were set as follows: minimal stack depth of 3, up to two mismatches within a locus at the individual level, up to four mismatches allowed when merging primary with secondary reads, up to six mismatches when merging tags from different specimens, removal of highly repetitive RAD tags. Out of this library, we used subsets of RAD tags with up to three SNPs, up to four SNPs, or exactly one SNP for further analyses in the Stacks v 1.34 program *populations* (Table 2). The read depth parameter is applied to alleles in the *denovo* and to individuals in the *populations* program (Stacks Manual p.24, 26). In order to avoid an artifactual surplus of real and erroneous homozygotes, we set the minimum read depth in *populations* to twice its value in *denovo* (i.e., to 6).

**Table 2:** Matrices of SNP data. Filtering setting in Stacks: –*p*: minimal number of populations required to be scored for each SNP; ‐*n*: maximum number of SNPs per locus; alleles: maximum number of alleles per locus. In all cases, only the first SNP of each locus is included

Data matrix	Populations included	‐*p*	‐*n*	Alleles	Unlinked SNPs	Analysis	Missing data (%)			
							Avg	*SD*	Min	Max
1	6	6	4	8	529	Structure	50	20	21	89
1a	6	6	1	2	151	Descriptive statistics, PCA and IBS	49	20	19	91
2	5	2	3	6	2,888	Structure	66	13	42	89
2a	5	2	1	2	1,319	Descriptive statistics, PCA and IBS	67	13	42	88

The amount of missing data is proportional to the genetic distance between taxa (Huang & Knowles, 2014). Preliminary analyses in Structure showed that the parameter setting that requires each locus to be present in at least two populations (‐p 2) led to a surplus of missing loci in the two most distinct populations in Rekem and Vardø due to many loci being shared only between the more closely related southern Norwegian sites. This in turn led to a tendency to cluster Belgium and Vardø, which may be an artifact. Therefore, we performed two alternative analyses: Matrix 1 with the loci present in all six populations, and Matrix 2 without the samples from Vardø and the loci present in at least two of the other five populations (Table 2).

To create Structure input files for both matrices, we used whitelists of loci with 1–4 SNPs and no more than eight alleles (Matrix 1), or 1–3 SNPs and no more than six alleles (Matrix 2) with the *export_sql.pl* program. In the program *populations*, each locus was required to occur at least once in a population. With the parameter “–write_single_snp,” we retained only the first SNP of each locus, avoiding obviously linked markers in the data matrix. Matrix 1 contained 529 SNPs and Matrix 2 contained 2,888 SNPs. On average, the samples have 50% and 66% missing data in the respective matrices, with large differences between individual stoneflies in the amount of missing data (Table 2; data matrix available from digital repository). We checked that the resulting markers all had exactly two alleles. Clustering of individuals in Structure was performed as described above for the AFLP data.

For the calculation of statistics describing within‐population homogeneity in the *populations* program, we used whitelists of loci containing only a single SNP and two alleles, avoiding the inclusion of closely linked SNPs, for both all six populations and the set without Vardø. (In contrast with the preparation of an input file for Structure, the program offers no “write single SNP” functionality to perform these calculations on loci with more than one SNP.) With the filter settings as above, this resulted in a much reduced number of loci in matrices 1a and 2a (Table 2).

In addition to Structure, we analyzed individual genotypes by means of Patterson's principal component analysis (PCA) and clustering by identity by state (IBS) as implemented in the R package SNPRelate v. 1.2.0 (Zheng et al., 2012). The IBS clustering algorithm draws a dendrogram based on an individual dissimilarity measure (Z score) based on the mean dissimilarity between random clusters, computed from permutation of individuals (Zheng, 2013: 66 ff.). For the PCA and IBS clustering, we produced two input files in vcf format with the *populations* program in Stacks. Here again, we used the same two subsets of loci and parameter settings as for the calculation of descriptive statistics, avoiding obviously linked SNPs (Table 2).

## 3 Results

### 3.1 Morphometry

**Figure 4:**
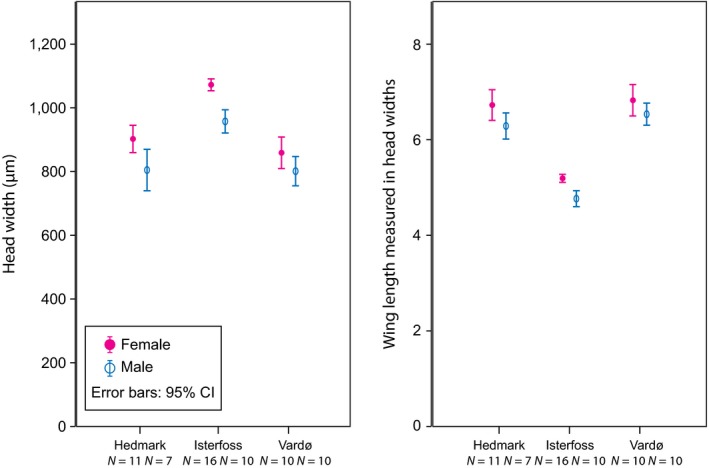
Head width (left graph) and forewing length scaled by head width (right graph) of four populations of *Leuctra hippopus* specimens. Hedmark pools samples from Ringsaker, Femundsenden, and Folldal. Females (left, filled pink symbol of each pair) tend to be larger and have longer wings than males

On average, females are larger than males, and they also have longer forewings relative to the width of the head (Figure 4, Table S2). *Leuctra hippopus* of both sexes from Isterfoss have significantly wider heads than those from either “other Hedmark” and Vardø (Figure 4). Likewise, the wings of both sexes are significantly shorter in Isterfoss than in the other Norwegian populations (Figure 4). Post hoc tests distinguish Isterfoss from either “other Hedmark” or Vardø with *p*‐values < .001 for head width and wing length in both sexes (Table S2). Our measurements show no difference between the specimens from Vardø and those from the “other Hedmark” group.

### 3.2 COI, 28S, and ITS sequences

**Figure 5:**
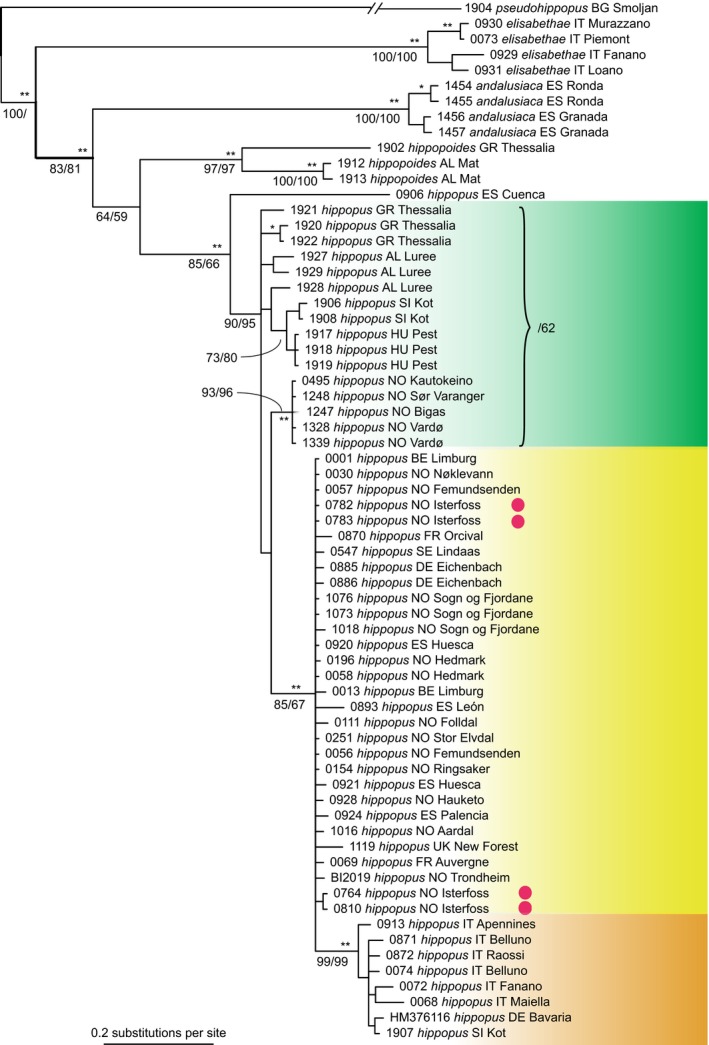
Bayesian tree of 67 specimens of *Leuctra hippopus* and outgroup species, based on a 636‐bp fragment of COI. Support values: *, ** indicate Bayesian posterior probability >.95 and >.99, respectively; MP and NJ bootstrap percentages are shown in this order separated by a slash. Bootstrap values for minor intraspecific nodes not shown. A possible eastern clade encompassing specimens from the Balkan and northernmost Norway was only retrieved in distance analysis

A COI phylogenetic tree was inferred from the 636‐bp‐long alignment of 67 specimens (Figure 5). Three major groups of haplotypes can be distinguished: a western clade stretching from central Spain to central Scandinavia, a central‐southern clade including Italy and the Alps, and an eastern group including the samples from Greece, Albania, Serbia, Slovenia, Hungary, and northernmost Norway. The central‐southern clade is a subclade of the western clade. There is no significant Bayesian or parsimony support for the monophyly of the eastern group, and only moderate support from distance analysis (bootstrap value 62). At the collecting site in Kot, Slovenia, an Italian and an eastern haplotype occur syntopically. A single specimen from Cuenca, central Spain, stands out as a fourth lineage. The four specimens from Isterfoss included in the COI matrix have two different haplotypes, both belonging to the western clade: one is widespread and also found in Huesca, north‐eastern Spain; the other differs by one nucleotide and has so far only been identified in Isterfoss. The partial 28S sequences (784 bp) show no intraspecific differentiation in *L. hippopus* (not shown), but the ITS sequences, displaying much variation in the form of indels, support the monophyly of the Norwegian population. In particular, the two ITS sequences from Isterfoss have the same ITS allele as the single sequence from Folldal (Figure S6).

### 3.3 AFLP

**Figure 6:**
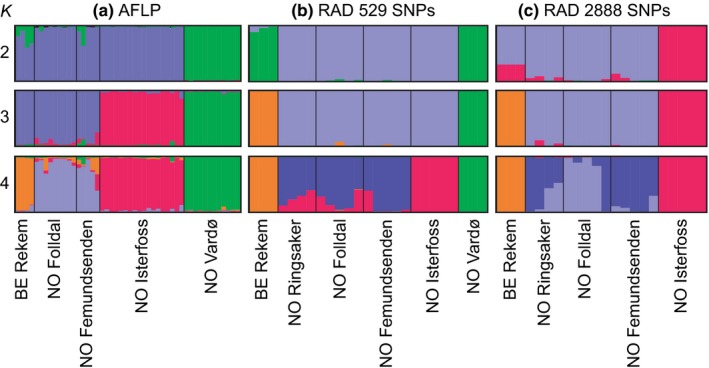
Cluster membership of *Leuctra hippopus* specimens and populations for *K* = 2—4, averaged over the same ten Structure runs, in three datasets: **(**a**)** 109 AFLP markers of 48 specimens from one Belgian and four Norwegian sites. **(**b**)** 529 RAD‐based SNPs of 25 specimens from Belgium and five Norwegian sites, where all SNPs are represented in each site. c. 2,888 RAD‐based SNPs of 22 specimens from Belgium and four Norwegian sites (excluding Vardø), where all SNPs are represented in at least two collecting sites

The analyses of the AFLP data in Structure show the highest posterior probability combined with a small standard deviation at *K* = 4, distinguishing (1) Vardø, (2) Isterfoss, (3) Folldal plus Femundsenden, and (4) Rekem (Figures 6a and S7). Evanno's measure ΔK reaches its highest value at *K* = 2, corresponding to the uppermost level of structuring in the dataset and separating Vardø from the West European populations. The AFLP data suggest that the distinction between Isterfoss and its geographical neighbors is larger than between the Belgian and the south Norwegian populations.

Clustering specimens or populations with the same data matrix in BAPS yielded three clusters, both with and without spatial information priors, if the maximum number of clusters *K* was set as 20: (1) Vardø, (2) Isterfoss, and (3) Folldal plus Femundsenden plus Rekem, corresponding to the Structure analysis with *K* = 3 (Figure 6a). With *K* set as 2, BAPS also distinguishes between Vardø and the western populations. BAPS did not detect admixture among the three clusters. The Voronoi tessellation graph (Figure S8) visualizes that the Isterfoss population differs from the neighboring populations.

### 3.4 RAD‐based SNPs

Table 3 gives descriptive data for the 151 unlinked SNPs represented in all six populations (Matrix 1a); Table 4 for the 1,319 unlinked SNPs each scored in at least two of the five West European populations (Matrix 2a). The complete statistics summary output of the *populations* program is available as supporting data. While the number of specimens included in the RAD library varies from three to five per population, the effective number scored per locus lies between 1.2 and 3.1. Measures of within‐population genetic variation (the number of polymorphic sites, frequency of the minor allele, and π) all show that Isterfoss and Vardø are more homogeneous than the other populations. The variation in Vardø may be underestimated due to its smaller sample size; Isterfoss, on the other hand, has the highest number of specimens scored per SNP of all six populations. The number of private alleles shows that Vardø is most distinct from the other populations, followed by Rekem. The four populations in Hedmark have similar numbers of private alleles.

**Table 3:** Descriptive statistics of 151 unlinked SNPs, each scored in all six populations, both within‐population variant and fixed sites

	Num Indv	Polymorphic Sites (%)	*Q*	Var (*Q*)	π	Var (π)	Private (%)
Rekem	1.91	0.34	0.0012	0.0005	0.0019	0.0012	30
Ringsaker	2.09	0.25	0.0009	0.0004	0.0014	0.0009	4
Femundsenden	2.06	0.24	0.0008	0.0003	0.0013	0.0008	10
Folldal	2.47	0.28	0.001	0.0004	0.0015	0.0009	12
Isterfoss	3.04	0.14	0.0004	0.0001	0.0007	0.0003	10
Vardø	1.21	0.05	0.0002	0.0001	0.0003	0.0002	34

Num Indv, average number of individuals per SNP; *Q*, frequency of the minor allele (1‐P); π, estimate of nucleotide diversity based on pairwise number of nucleotide differences; Var, variance; Private, percentage of the total number of 91 private alleles found in each population.

**Table 4:** Descriptive statistics of 1,319 unlinked SNPs each scored in at least two of the five West European populations, variant and fixed sites

	Num Indv	Polymorphic sites (%)	*Q*	Var (*Q*)	π	Var (π)	Private (%)
Rekem	1.61	0.26	0.0010	0.000	0.0016	0.0010	40
Ringsaker	1.82	0.31	0.0012	0.001	0.0019	0.0012	18
Femundsenden	1.69	0.23	0.0009	0.000	0.0014	0.0010	11
Folldal	2.01	0.27	0.0010	0.000	0.0016	0.0010	16
Isterfoss	2.68	0.19	0.0006	0.000	0.0010	0.0006	15

Num Indv, average number of individuals per SNP; *Q*, frequency of the minor allele (1‐P); π, estimate of nucleotide diversity based on pairwise number of nucleotide differences; Var, variance; Private, percentage of the total number of 825 private alleles found in each population.

The Structure analysis of SNP Matrix 1 including all six populations shows the likelihood of the clustering and Evanno's ΔK criterion peaking at *K* = 3 (Figure S9). The populations in Vardø and Belgium are distinguished first and the one in Isterfoss at *K* = 4 (Figures 6b and S9). At higher values of *K*, the specimens from Ringsaker, Folldal, and Femundsenden are subdivided, but no cluster corresponding to any of these collecting sites is formed (Figure S9). For Matrix 2 (without Vardø), with each locus being represented in at least two of the five West European populations, the highest value of ΔK corresponds to *K* = 3 (Figures 6c and S10), identifying the Belgian samples, Isterfoss, and the remaining sites from Hedmark county as separate clusters. When higher values of *K* are set, the Isterfoss cluster remains unchanged while the other clusters become subdivided. The histograms in Figures 6, S9 and S10 illustrate that the Isterfoss is more homogeneous than the other populations from southern Norway.

**Figure 7:**
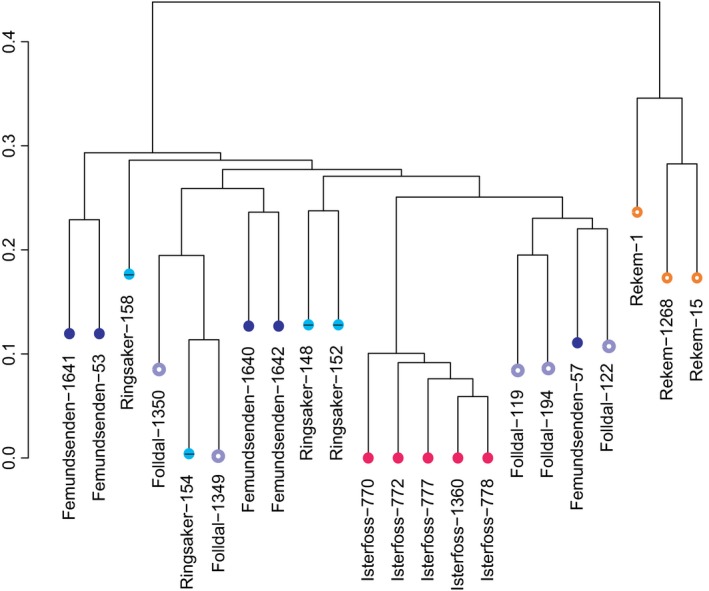
Identity by State clustering of 22 Belgian (Rekem) and south Norwegian *Leuctra hippopus* specimens based on 1,319 SNPs, each SNP being scored in at least two collecting sites. The scale bar indicates dissimilarity on a scale from 0 to 1

PCA and IBS clustering of the two smaller data matrices 1a and 2a (Table 2) yielded congruent results. In PCA, when all six populations are included, the first five eigenvectors of the PCA mostly distinguish the specimens from Vardø and Belgium from each other and from the south Norwegian ones (Figures S11–S13). Eigenvector 6 (5.4%) distinguishes Isterfoss from the other samples (Figure S13). In the PCA of the matrix without Vardø, eigenvector 1 (15.0%) shows the five western populations as adjacent clusters. This is the only analysis that distinguishes Ringsaker, Folldal, and Femundsenden from each other, albeit not sharply, and suggests that Folldal resembles Isterfoss most (Figure S14). Isterfoss forms a clearly distinct cluster in eigenvector 3 (Figure S15). The IBS clustering dendrograms based on the two data matrices are largely congruent with each other (Figure 7 and S16). The Belgian and the south Norwegian clusters together form a western cluster relative to specimens from Vardø. Within the south Norwegian cluster, only Isterfoss constitutes a distinct cluster. The Isterfoss population is both homogeneous and characterized by a number of shared alleles. In the IBS clustering of Matrix 1a with 151 SNPs, the three specimens that have most missing data (75–91%) end up in unexpected positions (Figure S16). This is an artifact that does not occur if, due to different data filtering, the dataset is slightly larger (data not shown).

## 4 Discussion

Both the morphometric measurements and genomewide sampled molecular data from AFLP and SNP confirm the distinctness of the Isterfoss population of *L. hippopus*. These genomewide sampled data as well as the COI and ITS sequences show that the Isterfoss population is part of a larger southern Scandinavian lineage. The distinctness of the population of the Isterfoss rapids is therefore due to local evolution, rather than a different origin. Analyses of AFLPs in Structure and BAPS and of SNPs in Structure detected little or no admixture between Isterfoss and nearby populations. The same analyses as well as IBS clustering of SNPs failed to distinguish between specimens from Ringsaker, Folldal, and Femundsenden, three localities 80–90 km from each other and belonging to two different major drainage basins. These results show that the Isterfoss population is really genetically isolated, as opposed to the three other, typical southern Norwegians sites, which the same methods cluster into a single, more diverse population. However, PCA of one of the SNP data matrices yielded a weak signal that Folldal is most, and Ringsaker least, similar to Isterfoss. This concurs with finding that Folldal and Isterfoss, but not Femundsenden, share the same ITS allele.

Measures of within‐population genetic variation show that Isterfoss displays a reduced genotypic variation, as expected for a small and isolated population. Little variation was also found in Vardø. Although the low scores of Vardø may be confounded by small sample size (some samples had relatively few loci scored), the genetic homogeneity of this coastal population is plausible. The collection site in Vardø is situated along a c. 10‐km‐long river on a peninsula on the northernmost edge of the continent (site FinLoc106 in Ekrem et al., 2012). Therefore, immigration is likely to contribute very little to local genetic variation in comparison with the collecting sites in southern Norway which are part of a large drainage basin.

### 4.1 Ecological speciation

The Isterfoss population of *L. hippopus* illustrates the process of ecological speciation. It can be discussed whether the current Isterfoss population of *L. hippopus* should be considered as a distinct species. Ultimately, this is a matter of preference and practical considerations (see Notes on taxonomy and phylogeography, Appendix S2). Scientifically, our interest is in demonstrating speciation by temporal isolation as an evolutionary process. Adaptation to the Isterfoss rapids is both morphological and phenological. The Isterfoss population is morphologically distinct from the other Norwegian populations in having wider heads and relatively short wings. The wider heads can be an expression of overall larger size, a more compact build or—most likely—a combination of both. The whole body is “fairly long” (Lillehammer, 1976: 171), and probably slightly longer than that of typical Norwegian populations (Lillehammer, 1987). The short wings measured in units of head width are likely due to a combination of wing reduction and a more stocky body shape. Both may have an adaptive value in that they enhance the adult stoneflies' ability to resist wind and cling to the boulders on which they aggregate and mate (Ørmen, 1991: 13). Typical *Leuctra* are slender long‐winged insects and Isterfoss specimens blown away from their aggregation sites would be unlikely to reproduce. Typical *L. hippopus* adults hide in riparian vegetation and are much less exposed to wind.

Phenological adaptation to local environmental factors is reflected in the early emergence and short adult life span of the Isterfoss population. While the higher autumn and winter temperature of the Isterfoss rapids enables faster growth of the immature stages, the early emergence and short adult life span are likely to be adaptations to avoid spring snowmelt floods that threaten to splash or even cover the boulders during May. Typical populations of *L. hippopus* are not particularly exposed to flooding as they emerge after snow melt and peak discharge, live in the riparian zone, and are capable of flight. The hypothesis that the phenological characteristics of the Isterfoss population are driven by flood avoidance (Ørmen, 1991: 14) finds support in the long‐term discharge data that show a sudden rise in water level in the second half of May, when the local *L. hippopus* population has completed its adult life stage. This phenological adaptation gives rise to temporal isolation of the Isterfoss population from nearby conspecific populations. Temporal isolation (Coyne & Orr, 2004: 202 ff.; Rundle & Nosil, 2005) due to nonoverlapping reproductive periods between Isterfoss and neighboring populations is likely to be the major cause of the genetic distinctness. Even though temporal isolation is a probable cause of diversification in aquatic insects with synchronized emergence, few studies have been published on this topic (Dijkstra et al., 2014). The Isterfoss population of *L. hippopus* may be among the clearest examples.

Many stonefly species use species‐specific patterns of substrate‐born vibrational signals for mate finding (Boumans & Johnsen, 2015; Stewart & Sandberg, 2005), and for this reason, Ørmen (1991) investigated whether variation in mating signals could be a barrier against gene flow between conspecific populations. He recorded male mating calls from Isterfoss as well as a number of other sites in Norway (Ørmen, 1991: 22–25), but no differences between populations were found (described in more detail by Cleven, 1992 s.a. [1992]: 30–31). No female signals of *L. hippopus* have ever been recorded; hence, it is not certain whether vibrational communication plays a role in mate finding or mate acceptance in this species. However, it almost certainly plays no role in the Isterfoss population as adults aggregate in high densities on nonresonant boulders. Besides, mating experiments revealed no ethological barrier to mating between males from Isterfoss and females from a typical population from Oslo (Ørmen, 1991: 25, 45–46).

It can be argued that Isterfoss is an example of sympatric speciation, depending on the scale in time and place. Typical adult *L. hippopus* disperse primarily along streams, mostly upstream, with few traveling farther than 150 m inland from the stream banks (Kuusela & Huusko, 1996; Müller, 1973; Petersen et al., 1999, 2004). However, with the help of stable isotope tracing in a mark–capture experiment, Macneale et al. (2005) showed for the Nearctic species *Leuctra ferruginea* that a tiny proportion of the mature females travel perpendicular to the watercourse over at least 800 m. They estimated that enough individuals travel far enough to ensure genetic connectivity between drainage basins a few kilometers distant. We found a population of typical *L. hippopus* 8 km away from Isterfoss but may have overlooked the species in more nearby streams. We interpret the fact that no clear genetic differentiation was detected between Ringsaker, Folldal, and Femundsenden as an indication that these three populations are sympatric at the spatial and temporal scales relevant to speciation. As Isterfoss is located between Folldal and Femundsenden, these populations are also sympatric with Isterfoss.

As weather conditions vary from year to year, it cannot be excluded that the presence of adult *L. hippopus* in Isterfoss and nearby streams overlaps in some years. The Isterfoss population does not fly, which may have contributed to its genetic isolation. Any admixture would result from nymphs drifting to or away from Isterfoss, or from adults flying to Isterfoss. This raises the question whether postzygotic isolation plays a role. Ørmen performed preliminary small‐scale mating experiments with males from Isterfoss and females from a locality near Oslo (150 m a.s.l.), which emerge at the same time of the year. In addition to a number of infertile matings, he reports a single female producing only 20 eggs of which nine hatched; the first‐instar nymphs died for unknown reasons (Ørmen, 1991: 60). Typically, a *L. hippopus* female lays hundreds of eggs of which >80% hatch if incubated under favorable conditions (Elliott, 1987). Ørmen's results are suggestive of intrinsic postzygotic isolation, but any conclusion must await a larger‐scale experiment with controls of same‐site matings. Because the Isterfoss population is adapted to its environment in several ways, we expect that hybrids would suffer reduced viability either in Isterfoss or in a typical *L. hippopus* habitat (extrinsic postzygotic isolation).

The Isterfoss population must have diverged locally from other south Norwegian populations in the 10,000 years after the last glaciation. The four collecting sites in Hedmark (Ringsaker, Folldal, Femundsenden, and Isterfoss) have similar numbers of private alleles in the RAD‐based SNPs. That only the specimens from Isterfoss form a distinct cluster appears to be due to their similarity, that is, more private and nonprivate alleles being shared among the specimens, rather than to their strong genetic divergence from the other Hedmark populations. Our interpretation is that genomewide allelic divergence is primarily a function of time and that the different south Norwegian populations are of similar age. The divergence of the Isterfoss population in morphology and life cycle characteristics does imply genetic changes, but these are likely to be few relative to the wide sampling of RAD loci.

While the Isterfoss rapids are now inhabited by an atypical form of *L. hippopus*, it has not in recent times been recolonized by the typical form, even though this stonefly is common in all suitable habitats in Scandinavia. The habitat characteristics of the rapids are so different from the species' typical habitat with riparian vegetation that it may not be a suitable habitat for the typical *L. hippopus*. So how did this species colonize the Isterfoss rapids in the first place? A possible explanation is gradual habitat isolation, similar to cospeciation of parasites or pollinators and their hosts (Coyne & Orr, 2004: 191). We propose that *L. hippopus* colonized Isterfoss at a time when it was more similar to other streams in the vicinity. Riverine erosion of the moraine bottom changed a broader and shallower stream into a narrower fast running stream flanked by flats of deposited boulders. In this process, *L. hippopus* persisted and became trapped in the two emerging rows of large boulders flanking the main stream, while the boulder flats became unsuitable habitat, possibly because of insufficient water flow.

About one‐fifth of the European stonefly species are micro‐endemics restricted to small isolated mountain ranges (Graf et al., 2009: 9). In particular, the families Nemouridae and Leuctridae include many short‐range endemics (Aubert, 1959; Graf et al., 2009: 75–82; Vinçon & Ravizza, 2000). It is reasonable to assume that processes of ecological speciation similar to that in Isterfoss together with the generally restricted dispersal capacity of stoneflies explain this high incidence of endemism. Intraspecific genetic differentiation in different ranges of the same stream was found in some studies (Alp et al., 2012; Watanabe et al., 2014; Wofford et al., 2005), and there are several examples of congeneric species of aquatic insects inhabiting adjacent ranges. Differential longitudinal distribution and adaptation to the local environment is probably a common speciation mechanism in lotic invertebrates. Our study of the population in the Isterfoss rapids, which were glaciated until approximately 10,000 years ago, shows that genetic isolation and adaptation to very local circumstances can take place in sympatry over a relatively short time period.

## Conflict of Interest

None declared.

## Data Accessibility

Main characteristics of the samples included in this study are provided in Table S1, together with GenBank accession numbers for the associated DNA sequences; additional details are available from the BOLD online database (http://www.boldsystems.org/). Appendix S1 provides methodological details. Morphometric data are provided as Table S2. RAD tag reads filtered for duplications by means of the clone_filter program in Stacks are archived in SRA (Bioproject ID PRJNA348404, Accession No SRP091758), while raw data matrices of both AFLP and RAD‐based SNPs used in our analyses, and the “Sumstats” statistics output of the Stacks program are archived in the Dryad Digital Repository (http://dx.doi.org/10.5061/dryad.231b9).
