# Supplementary material for: Ecological speciation by temporal isolation in a population of the stonefly Leuctra hippopus (Plecoptera, Leuctridae)
Source: Ecol Evol. 2017 Feb 10;7(5):1635–49. doi: 10.1002/ece3.2638 (PMC5330929; doi:10.1002/ece3.2638)
Supplement: Supplementary file 1 [file ECE3-7-1635-s001.doc]

# Supplementary figures S1–S16


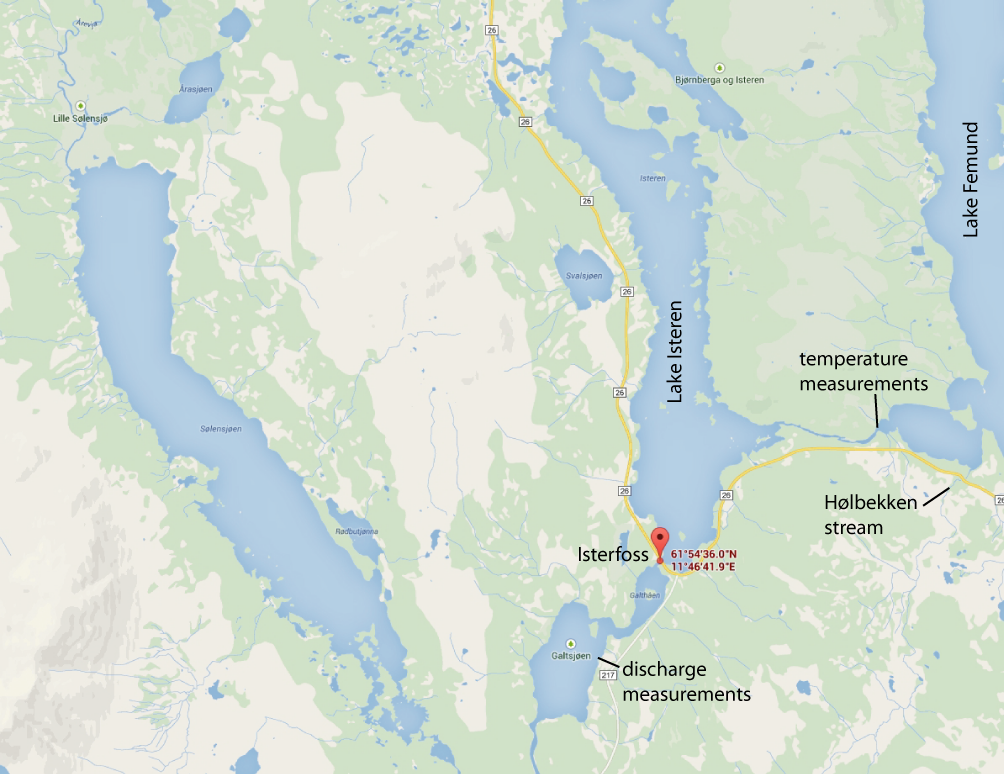


**Figure S1. Location of the Isterfoss rapids (61°54.599' N 11°46.698' E.), the Hølbekken stream (61°55.146'N 11°55.674'E) and hydrological measurement stations: outflow of Lake Femund (61°56.094' N 11°52.034' E) and shore of Lake Galten (61°53.479' N 11°45.227' E). Map from Google Maps.**


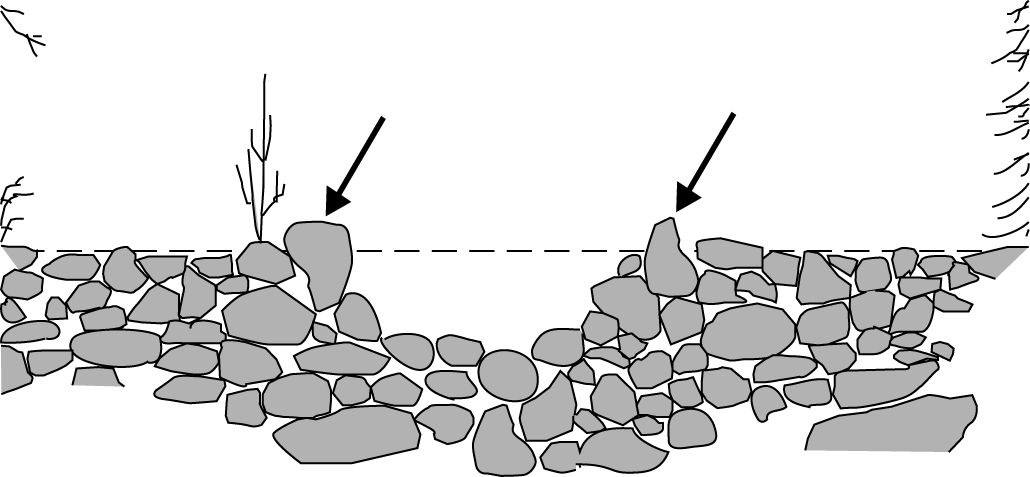


Figure S2. Sketch of transverse river bed at Isterfoss. Adult *L. hippopus* occur on the large boulders along the stream on either side (arrows) from the second half of April to the second half of May. The dotted line indicates the water level in that period of the year.

**
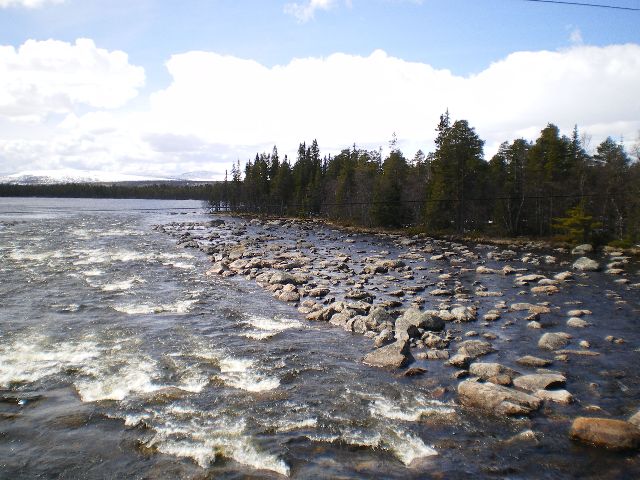
**

**Figure S3. Western half of Isterfoss photographed from the bridge on 8 May 2012.**


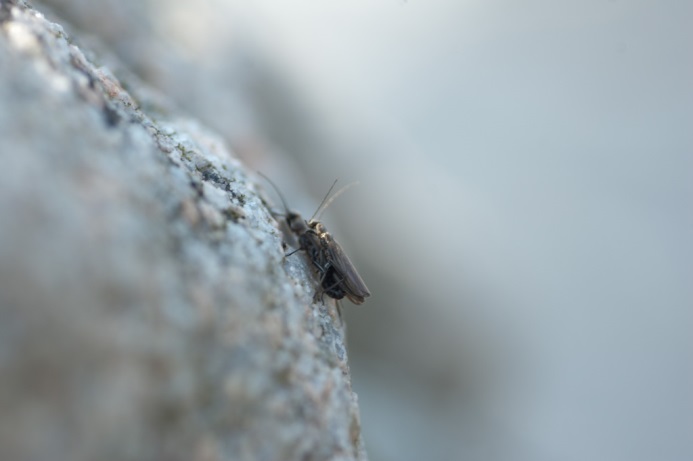
**Figure S4. Mating pair of *Leuctra hippopus* on one of the central boulders in Isterfoss on 8 May 2012. Lichen species growing on the boulders and on which *L. hippopus* may feed were identified from photographs by Einar Timdal: *Xanthoparmelia conspersa*, *Rhizocarpon geographicum*, *Umbilicaria polyphylla* (?), *Parmelia saxatilis*, *Umbilicaria deusta*, *Parmelia saxatilis*, *Rhizocarpon badioatrum*, *Physcia dubia*, *Umbilicaria vellea*.**


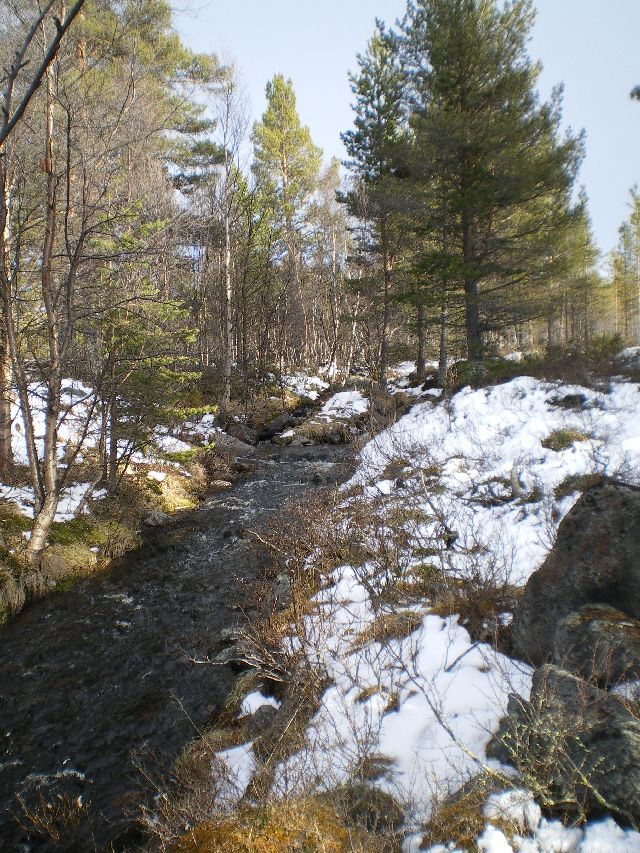
**Figure S5. Typical habitat of *Leuctra hippopus* in Norway: the Hølbekken stream in Femundsenden, photo taken on 7 May 2012. Adults emerge from this stream by the end of May.**


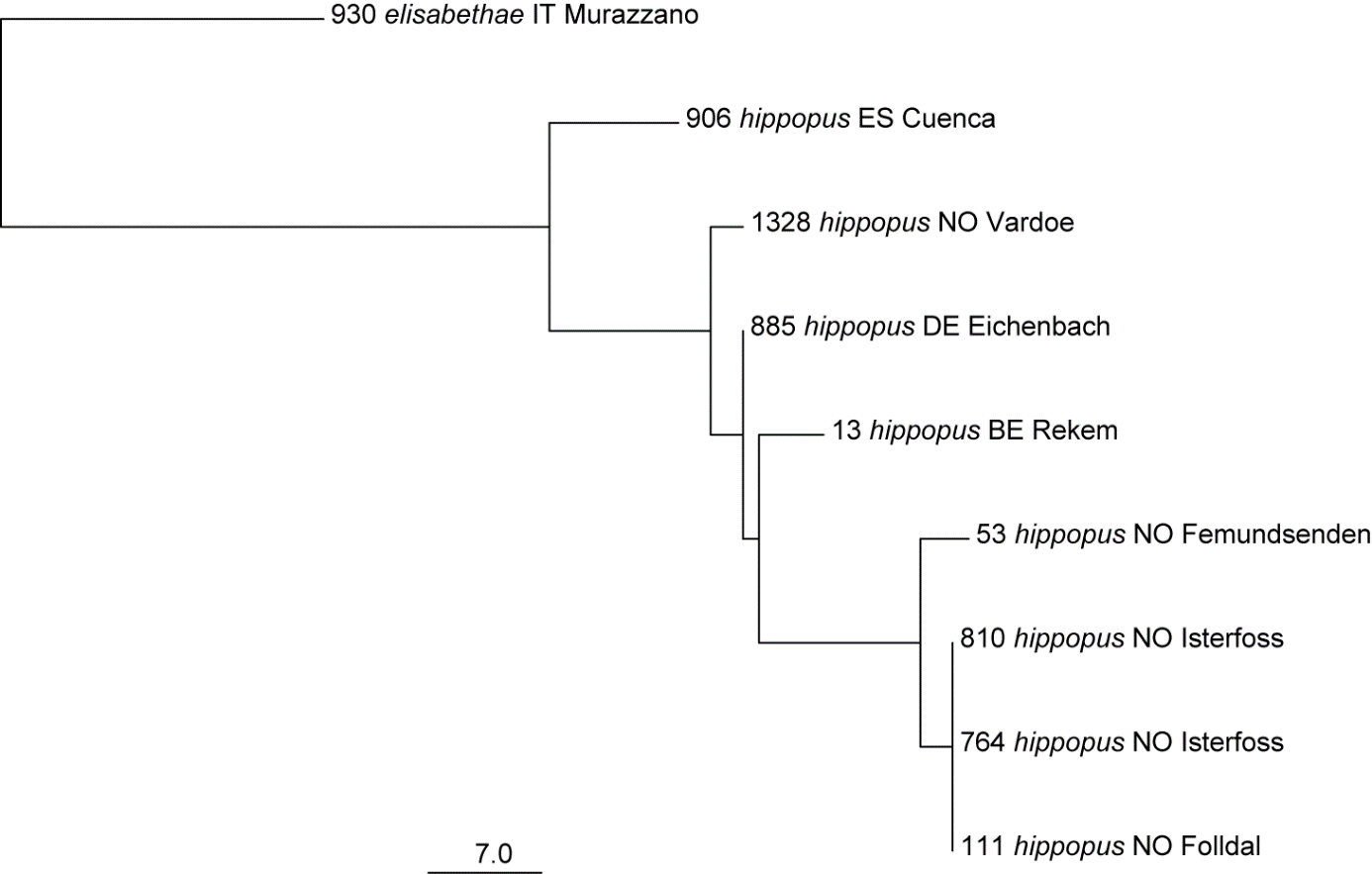


**Figure S6. Most parsimonious tree diagram of nine ITS sequences (931 bp) with gaps treated as a fifth state. Note that this graph gives a skewed image of the variation, as longer indels yield longer branches than short indels, even though both may be due to a single mutation event.**


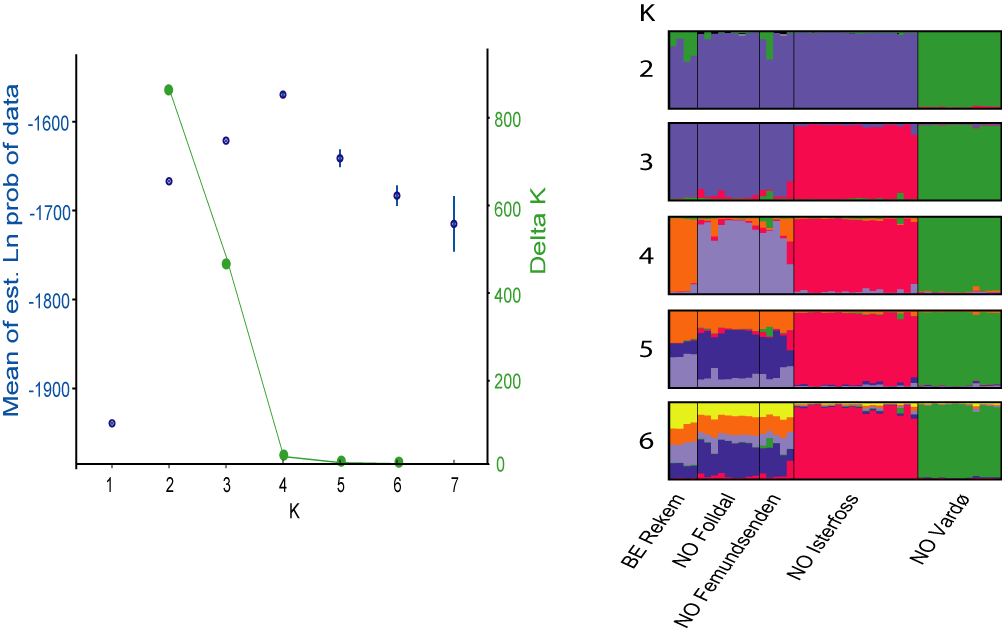


Figure S7. Structure clustering of 48 *Leuctra hippopus* specimens from one Belgian and four Norwegian sites based on 109 AFLP markers. Left: Posterior likelihood and Evanno’s ΔK criterion of 2–7 clusters based on ten Structure runs. Right: Cluster membership of each specimen and population for K=2—6, averaged over the same ten Structure runs.


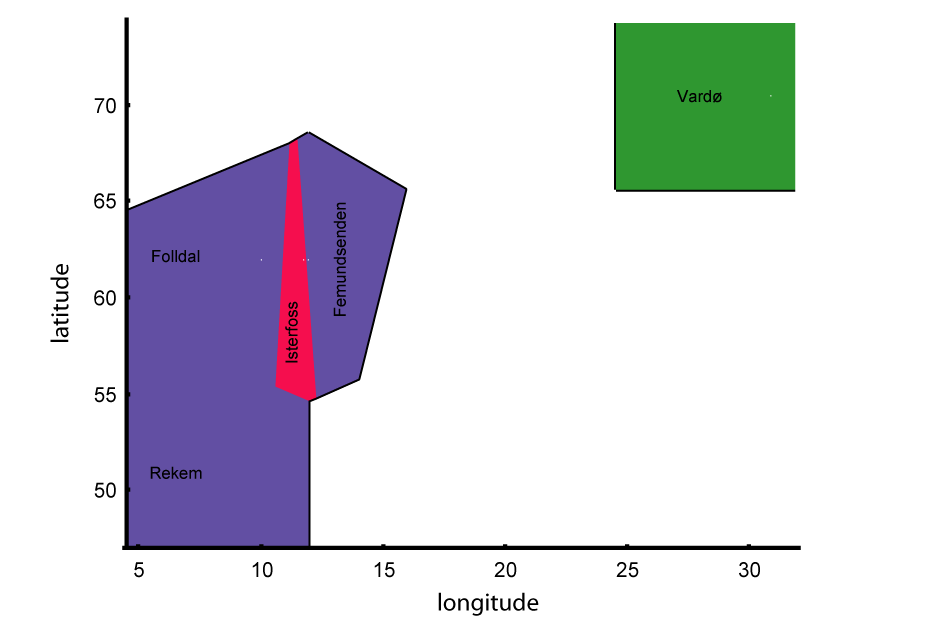


**Figure S8. Voronoi tessellation graph showing the clustering of 48 samples based on 109 AFLP markers in BAPS. Three clusters are recognised. Isterfoss is recognised as a distinct cluster in spite of being geographically between Folldal and Femundsenden.**


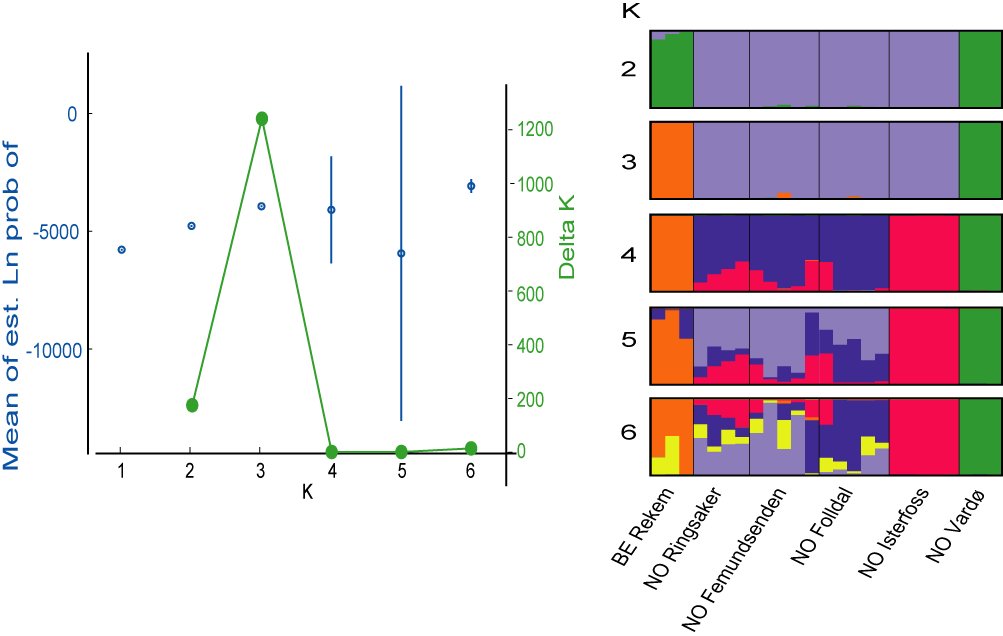


Figure S9. Clustering 25 *Leuctra hippopus* specimens from Norway and Belgium based on 529 RAD-based SNPs represented in all six collecting sites. Left: Posterior likelihood and Evanno’s ΔK criterion of 2–6 clusters based on ten Structure runs. Right: Cluster membership of each specimen and population for K=2—6, averaged over the same ten Structure runs.


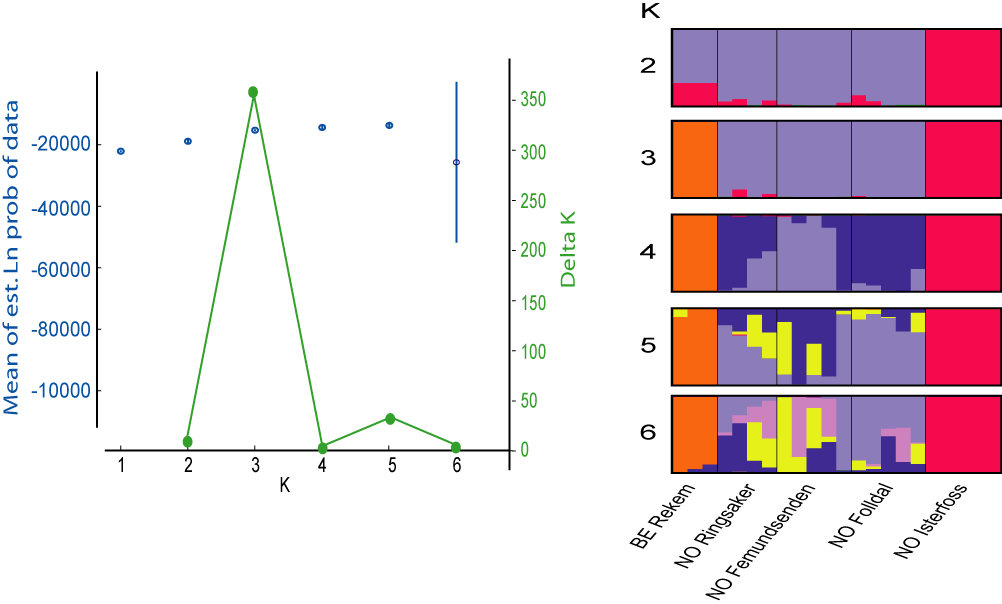


Figure S10. Clustering 22 *Leuctra hippopus* specimens from Norway and Belgium based on 2888 RAD-based SNPs represented in at least two of five collecting sites. Left: Posterior likelihood and Evanno’s ΔK criterion of 2–6 clusters based on ten Structure runs. Right: Cluster membership of each specimen and population for K=2—6, averaged over the same ten Structure runs.


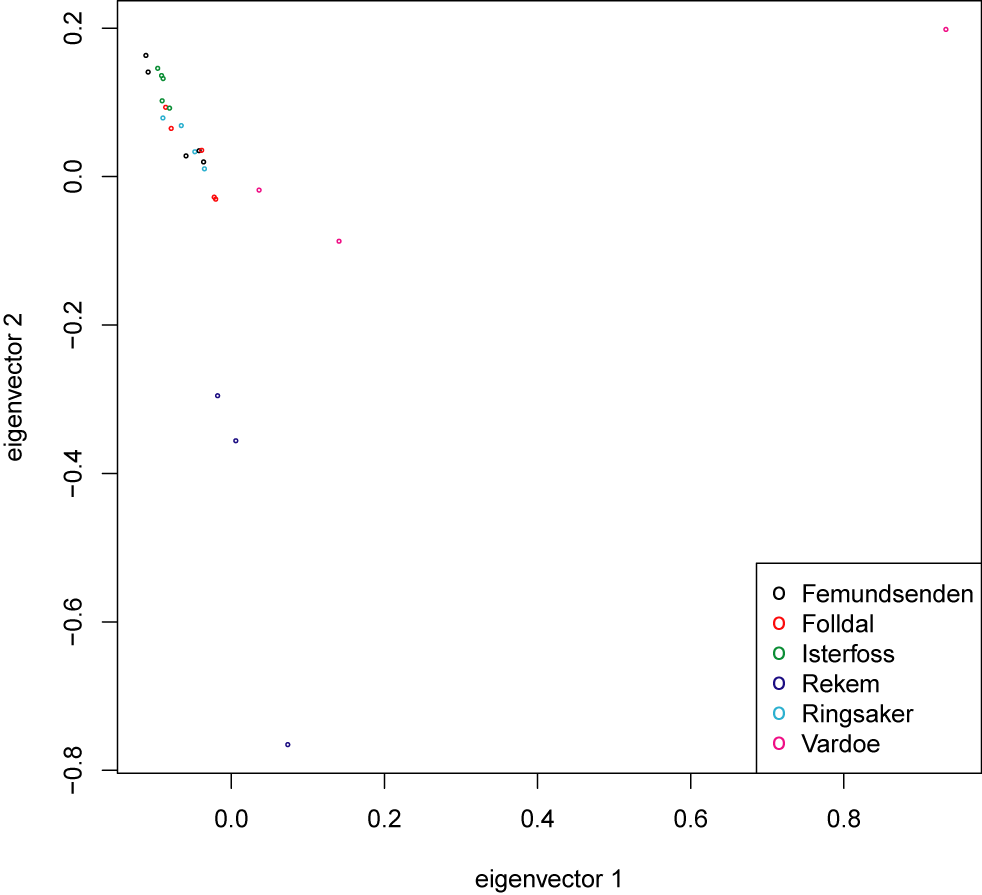


**Figure S11. Principal Component Analysis of 25 *Leuctra hippopus* specimens based on 183 unlinked SNPs represented in all six collecting sites in Belgium and Norway, eigenvectors 1 (23.2%) and 2 (10.4%).**


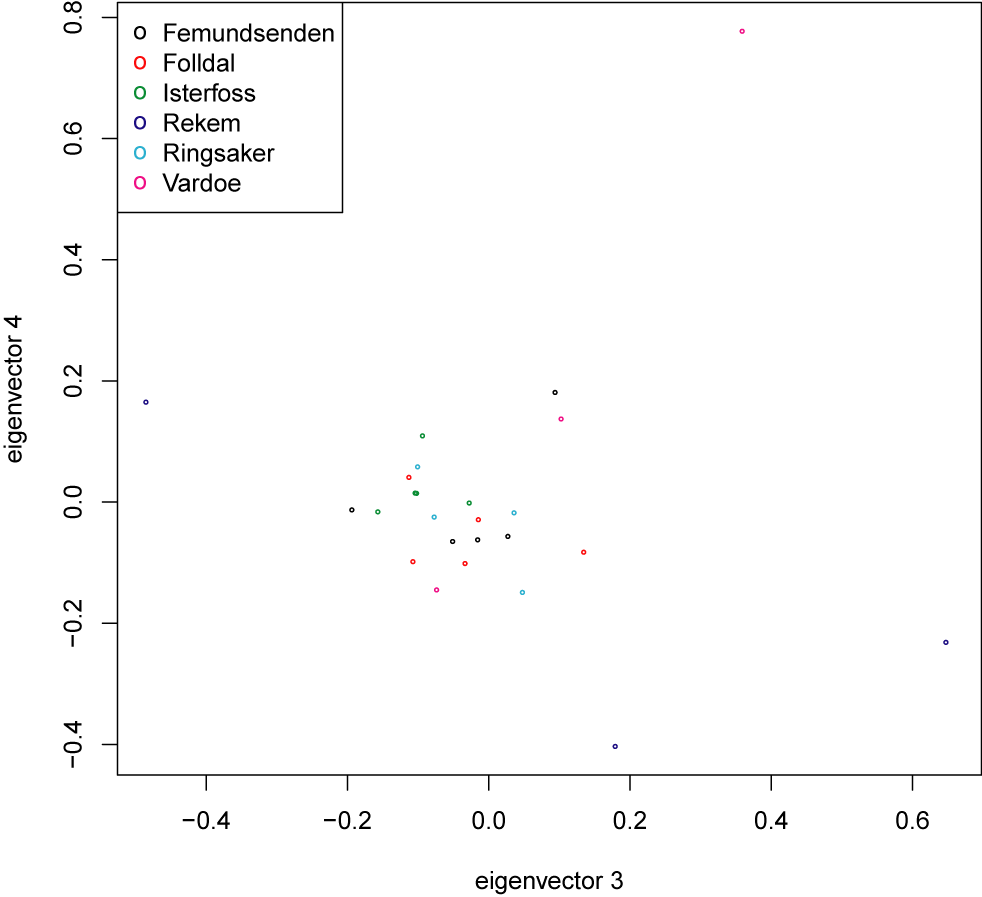


**Figure S12. Principal Component Analysis of 25 *Leuctra hippopus* specimens based on 183 unlinked SNPs represented in all six collecting sites in Belgium and Norway, eigenvectors 3 (7.4%) and 4 (6.2%).**


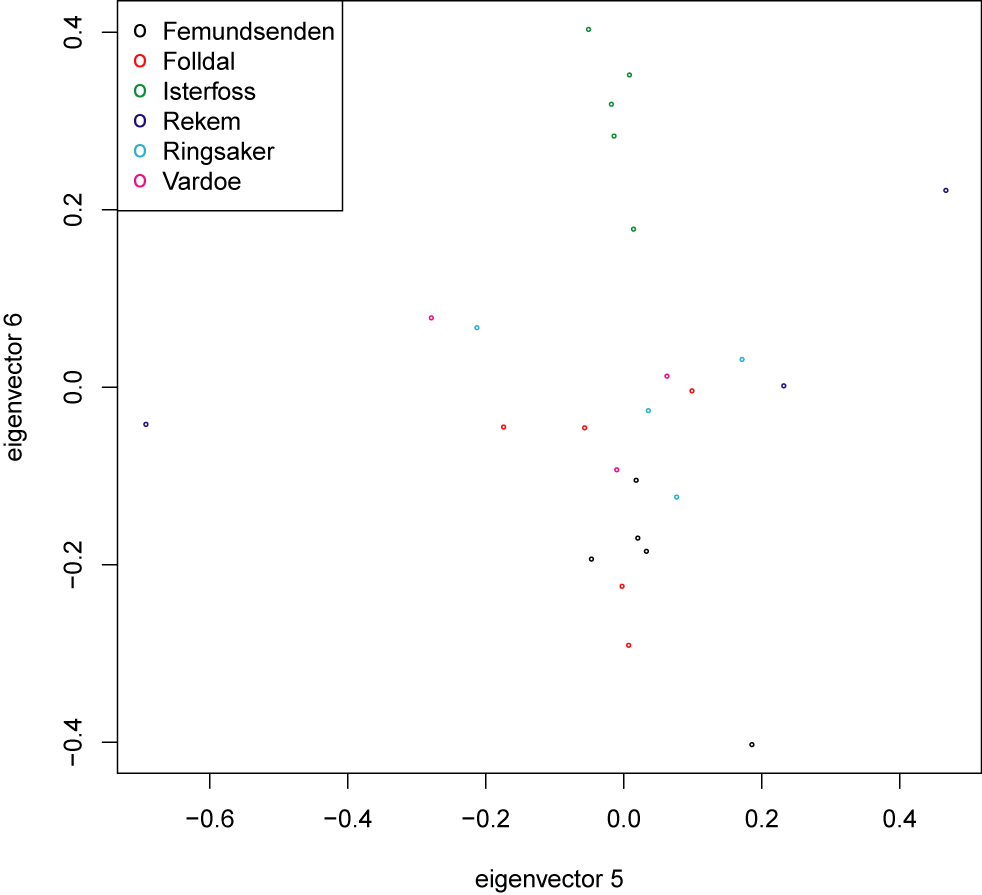


**Figure S13. Principal Component Analysis of 25 *Leuctra hippopus* specimens based on 183 unlinked SNPs represented in all six collecting sites in Belgium and Norway, eigenvectors 5 (5.7%) and 6 (5.4%).**


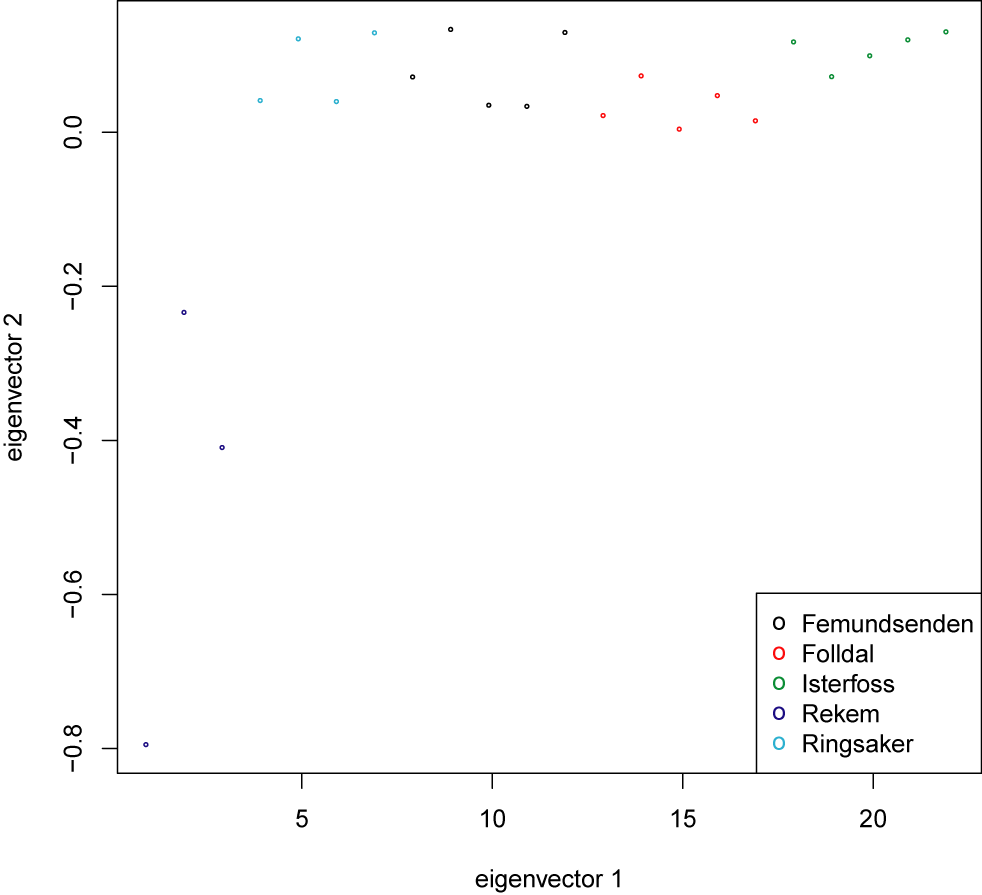


**Figure S14. Principal Component Analysis of 22 *Leuctra hippopus* specimens based on 1377 unlinked SNPs represented in at least two collecting sites in Belgium and Norway, eigenvectors 1 (15.0%) and 2 (10.4%).**


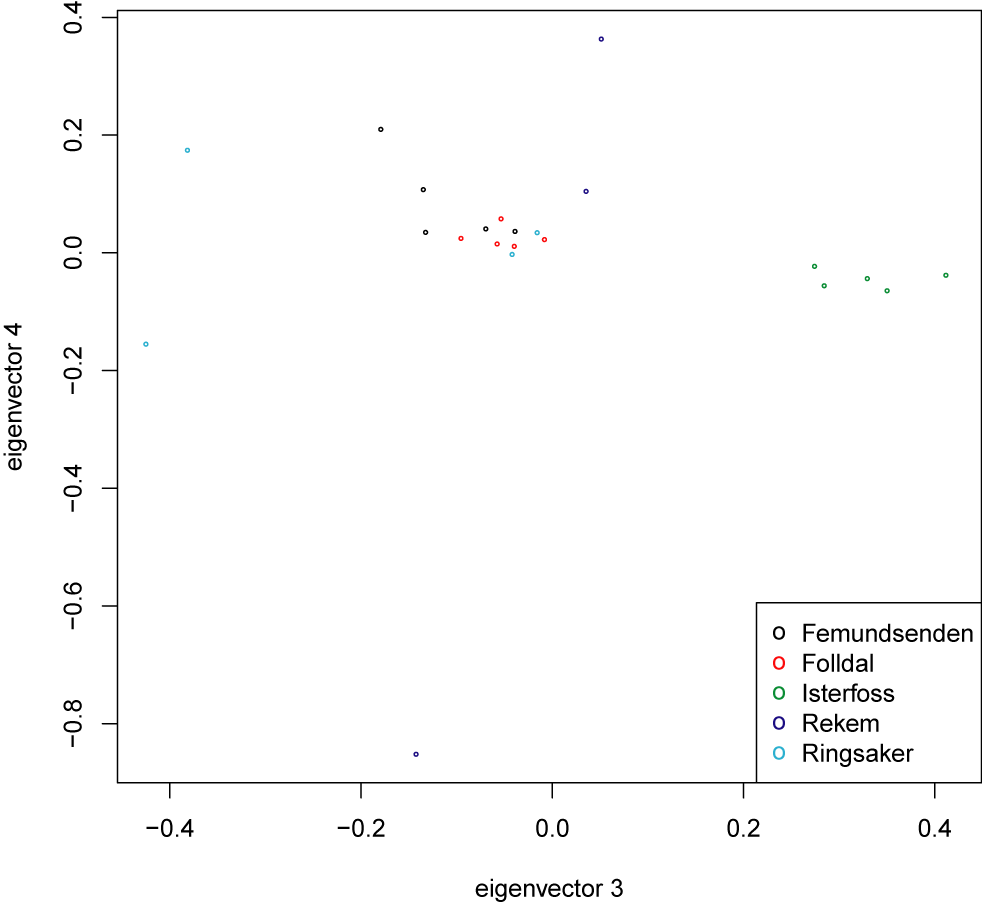


**Figure S15. Principal Component Analysis of 22 *Leuctra hippopus* specimens based on 1377 unlinked SNPs represented in at least two collecting sites in Belgium and Norway, eigenvectors 3 (8.4%) and 4 (8.0%).**


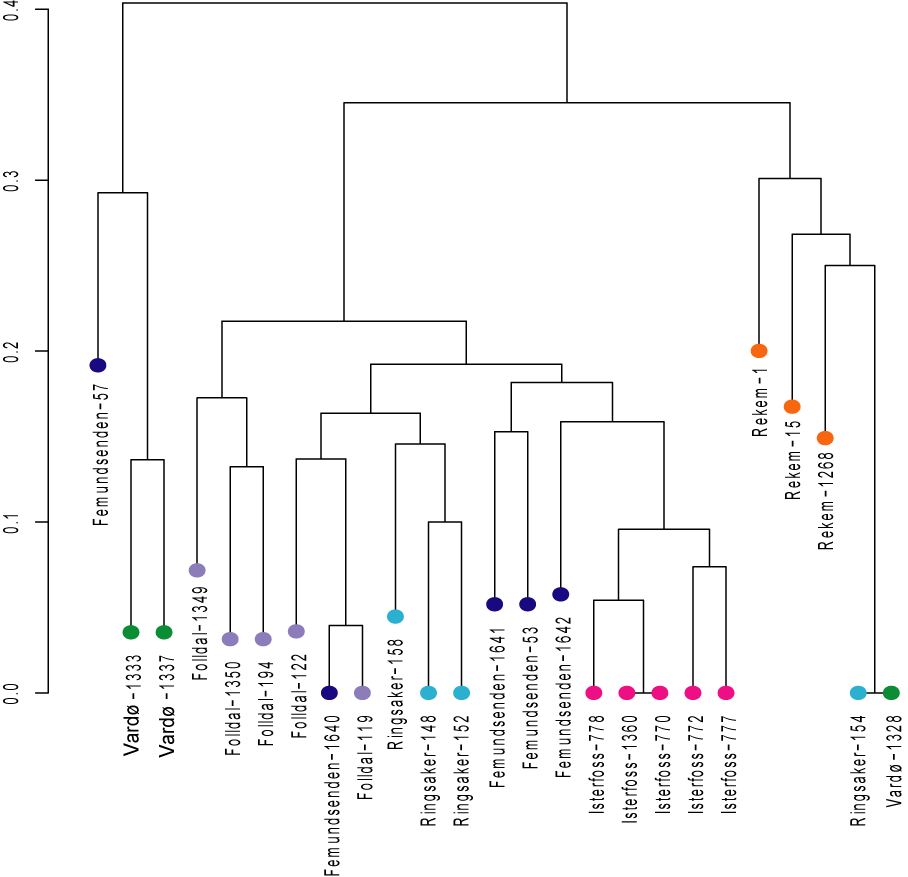


**Figure S16. Identity by State clustering of 25 *Leuctra hippopus* specimens based on 151 SNPs represented in all six collecting sites in Belgium and Norway. Specimens Femundsenden-57, Ringsaker-154 and Vardoe-1328 have the highest proportion missing data (75–91%).**
