# Supplementary material for: Ecological speciation by temporal isolation in a population of the stonefly Leuctra hippopus (Plecoptera, Leuctridae)
Source: Ecol Evol. 2017 Feb 10;7(5):1635–49. doi: 10.1002/ece3.2638 (PMC5330929; doi:10.1002/ece3.2638)
Supplement: Supplementary file 2 [file ECE3-7-1635-s002.docx]

| **Table S1. *Leuctra* specimens used in molecular analyses.** | | | | | | | | |
| --- | --- | --- | --- | --- | --- | --- | --- | --- |
| cat. nr. | species | country | location | COI | 28S | ITS | AFLP | RAD |
| 31456 | *andalusiaca* | ES | Granada | KT874642 |  |  |  |  |
| 31457 | *andalusiaca* | ES | Granada | KT874643 | KX536635 |  |  |  |
| 31454 | *andalusiaca* | ES | Ronda | KT874651 | KX536637 |  |  |  |
| 31455 | *andalusiaca* | ES | Ronda | KT874623 |  |  |  |  |
| 30929 | *elisabethae* | IT | Fanano | KT874621 |  |  |  |  |
| 30931 | *elisabethae* | IT | Loano | KT874677 | KX536639 |  |  |  |
| 30930 | *elisabethae* | IT | Murazzano | KT874667 |  | KT874691 |  |  |
| 30073 | *elisabethae* | IT | Piemont | KT874639 |  |  |  |  |
| 31912 | *hippopoides* | AL | Mat | KT874681 |  |  |  |  |
| 31913 | *hippopoides* | AL | Mat | KT874668 |  |  |  |  |
| 31902 | *hippopoides* | GR | Thessalia | KT874680 |  |  |  |  |
| 31927 | *hippopus* | AL | Luree | KT874637 |  |  |  |  |
| 31928 | *hippopus* | AL | Luree | KT874641 |  |  |  |  |
| 31929 | *hippopus* | AL | Luree | KT874631 |  |  |  |  |
| 30001 | *hippopus* | BE | Rekem | KF809176 | KF809177 |  | 1 | 1 |
| 30013 | *hippopus* | BE | Rekem | KT874624 |  | KT874684 |  |  |
| 30015 | *hippopus* | BE | Rekem |  |  |  |  | 1 |
| 30077 | *hippopus* | BE | Rekem |  |  |  | 1 |  |
| 30083 | *hippopus* | BE | Rekem |  |  |  | 1 |  |
| 31268 | *hippopus* | BE | Rekem |  |  |  | 1 | 1 |
| ZSMAQU 00011 | *hippopus* | DE | Bavaria | HM376116 |  |  |  |  |
| 30885 | *hippopus* | DE | Eichenbach | KT874671 |  | KT874692 |  |  |
| 30886 | *hippopus* | DE | Eichenbach | KT874653 |  |  |  |  |
| 30906 | *hippopus* | ES | Cuenca | KT874633 |  | KT874687 |  |  |
| 30920 | *hippopus* | ES | Huesca | KT874650 |  |  |  |  |
| 30921 | *hippopus* | ES | Huesca | KT874657 | KX536638 |  |  |  |
| 30893 | *hippopus* | ES | León | KT874618 |  |  |  |  |
| 30924 | *hippopus* | ES | Palencia | KT874673 |  |  |  |  |
| 30069 | *hippopus* | FR | Auvergne | KT874645 |  |  |  |  |
| 30870 | *hippopus* | FR | Orcival | KT874647 |  |  |  |  |
| 31920 | *hippopus* | GR | Thessalia | KT874655 |  |  |  |  |
| 31921 | *hippopus* | GR | Thessalia | KT874656 |  |  |  |  |
| 31922 | *hippopus* | GR | Thessalia | KT874620 |  |  |  |  |
| 31917 | *hippopus* | HU | Pest | KT874664 |  |  |  |  |
| 31918 | *hippopus* | HU | Pest | KT874678 |  |  |  |  |
| 31919 | *hippopus* | HU | Pest | KT874666 |  |  |  |  |
| 30913 | *hippopus* | IT | Apennines | KT874658 |  |  |  |  |
| 30074 | *hippopus* | IT | Belluno | KT874629 |  |  |  |  |
| 30871 | *hippopus* | IT | Belluno | KT874626 | KX536633 |  |  |  |
| 30072 | *hippopus* | IT | Fanano | KT874665 |  |  |  |  |
| 30068 | *hippopus* | IT | Maiella | KT874682 |  |  |  |  |
| 30872 | *hippopus* | IT | Raossi | KT874622 |  |  |  |  |
| 31016 | *hippopus* | NO | Aardal | KT874632 |  |  |  |  |
| 31247 | *hippopus* | NO | Bigas | KT874663 |  |  |  |  |
| 30052 | *hippopus* | NO | Femundsenden |  |  |  | 1 |  |
| 30053 | *hippopus* | NO | Femundsenden |  |  | KT874686 | 1 | 1 |
| 30056 | *hippopus* | NO | Femundsenden | KT874636 |  |  | 1 |  |

| cat. nr. | species | country | location | COI | 28S | ITS | AFLP | RAD |
| --- | --- | --- | --- | --- | --- | --- | --- | --- |
| 30057 | *hippopus* | NO | Femundsenden | KT874654 | KX536636 | KT874689 | 1 | 1 |
| 30058 | *hippopus* | NO | Femundsenden | KT874669 |  |  | 1 |  |
| 31640 | *hippopus* | NO | Femundsenden |  |  |  |  | 1 |
| 31641 | *hippopus* | NO | Femundsenden |  |  |  |  | 1 |
| 31642 | *hippopus* | NO | Femundsenden |  |  |  |  | 1 |
| 30111 | *hippopus* | NO | Folldal | KT874649 |  |  | 1 |  |
| 30119 | *hippopus* | NO | Folldal |  |  |  | 1 | 1 |
| 30120 | *hippopus* | NO | Folldal |  |  |  | 1 |  |
| 30121 | *hippopus* | NO | Folldal |  |  |  | 1 |  |
| 30122 | *hippopus* | NO | Folldal |  |  |  | 1 | 1 |
| 30194 | *hippopus* | NO | Folldal |  |  |  | 1 | 1 |
| 30196 | *hippopus* | NO | Folldal | KT874640 |  |  | 1 |  |
| 31349 | *hippopus* | NO | Folldal |  |  |  | 1 | 1 |
| 31350 | *hippopus* | NO | Folldal |  |  |  |  | 1 |
| 31351 | *hippopus* | NO | Folldal |  |  |  | 1 |  |
| 30928 | *hippopus* | NO | Hauketo | KT874662 |  |  |  |  |
| 30764 | *hippopus* | NO | Isterfoss | KT874646 |  | KT874688 | 1 |  |
| 30766 | *hippopus* | NO | Isterfoss |  |  |  | 1 |  |
| 30767 | *hippopus* | NO | Isterfoss |  |  |  | 1 |  |
| 30768 | *hippopus* | NO | Isterfoss |  |  |  | 1 |  |
| 30769 | *hippopus* | NO | Isterfoss |  |  |  | 1 |  |
| 30770 | *hippopus* | NO | Isterfoss |  |  |  |  | 1 |
| 30772 | *hippopus* | NO | Isterfoss |  |  |  |  | 1 |
| 30773 | *hippopus* | NO | Isterfoss |  |  |  | 1 |  |
| 30774 | *hippopus* | NO | Isterfoss |  |  |  | 1 |  |
| 30775 | *hippopus* | NO | Isterfoss |  |  |  | 1 |  |
| 30777 | *hippopus* | NO | Isterfoss |  |  |  |  | 1 |
| 30778 | *hippopus* | NO | Isterfoss |  |  |  |  | 1 |
| 30779 | *hippopus* | NO | Isterfoss |  |  |  | 1 |  |
| 30781 | *hippopus* | NO | Isterfoss |  |  |  | 1 |  |
| 30782 | *hippopus* | NO | Isterfoss | KT874670 |  |  | 1 |  |
| 30783 | *hippopus* | NO | Isterfoss | KT874634 |  |  | 1 |  |
| 30784 | *hippopus* | NO | Isterfoss |  |  |  | 1 |  |
| 30786 | *hippopus* | NO | Isterfoss |  |  |  | 1 |  |
| 30809 | *hippopus* | NO | Isterfoss |  |  |  | 1 |  |
| 30810 | *hippopus* | NO | Isterfoss | KT874630 |  | KT874685 | 1 |  |
| 30816 | *hippopus* | NO | Isterfoss |  |  |  | 1 |  |
| 30817 | *hippopus* | NO | Isterfoss |  |  |  | 1 |  |
| 31630 | *hippopus* | NO | Isterfoss |  |  |  |  | 1 |
| 30495 | *hippopus* | NO | Kautokeino | KT874648 |  |  |  |  |
| 30030 | *hippopus* | NO | Nøklevann | KT874659 |  |  |  |  |
| 30148 | *hippopus* | NO | Ringsaker |  |  |  |  | 1 |
| 30152 | *hippopus* | NO | Ringsaker |  |  |  |  | 1 |
| 30154 | *hippopus* | NO | Ringsaker | KT874644 |  |  |  | 1 |
| 30158 | *hippopus* | NO | Ringsaker |  |  |  |  | 1 |
| 31248 | *hippopus* | NO | Sør Varanger | KT874635 | KX536634 |  |  |  |
| 31018 | *hippopus* | NO | Sogn og Fjordane | KT874619 |  |  |  |  |
| 31073 | *hippopus* | NO | Sogn og Fjordane | KT874625 |  |  |  |  |
| 31076 | *hippopus* | NO | Sogn og Fjordane | KT874672 |  |  |  |  |

| cat. nr. | species | country | location | COI | 28S | ITS | AFLP | RAD |
| --- | --- | --- | --- | --- | --- | --- | --- | --- |
| 30251 | *hippopus* | NO | Stor Elvdal | KT874660 |  | KT874690 | 1 | 1 |
| BI2019 | *hippopus* | NO | Trondheim | KT874674 |  |  |  |  |
| 31328 | *hippopus* | NO | Vardø | KT874661 |  |  |  |  |
| 31329 | *hippopus* | NO | Vardø |  |  |  | 1 |  |
| 31330 | *hippopus* | NO | Vardø |  |  |  | 1 |  |
| 31331 | *hippopus* | NO | Vardø |  |  |  | 1 |  |
| 31332 | *hippopus* | NO | Vardø |  |  |  | 1 |  |
| 31333 | *hippopus* | NO | Vardø |  |  |  | 1 | 1 |
| 31334 | *hippopus* | NO | Vardø |  |  |  | 1 |  |
| 31335 | *hippopus* | NO | Vardø |  |  |  | 1 |  |
| 31336 | *hippopus* | NO | Vardø |  |  |  | 1 |  |
| 31337 | *hippopus* | NO | Vardø |  |  |  | 1 | 1 |
| 31338 | *hippopus* | NO | Vardø |  |  |  | 1 |  |
| 31339 | *hippopus* | NO | Vardø | KT874638 |  |  | 1 |  |
| 30547 | *hippopus* | SE | Lindås | KT874628 |  |  |  |  |
| 31906 | *hippopus* | SI | Kot | KT874675 |  |  |  |  |
| 31907 | *hippopus* | SI | Kot | KT874627 |  |  |  |  |
| 31908 | *hippopus* | SI | Kot | KT874679 |  |  |  |  |
| 31119 | *hippopus* | UK | New Forest | KT874676 |  |  |  |  |
| 31904 | *pseudohippopus* | BG | Smoljan | KT874683 |  |  |  |  |
| COUNT |  |  |  | 66 | 7 | 9 | 48 | 25 |
