# Supplementary material for: Ecological speciation by temporal isolation in a population of the stonefly Leuctra hippopus (Plecoptera, Leuctridae)
Source: Ecol Evol. 2017 Feb 10;7(5):1635–49. doi: 10.1002/ece3.2638 (PMC5330929; doi:10.1002/ece3.2638)
Supplement: Supplementary file 3 [file ECE3-7-1635-s003.docx]

**Table S2 Morphometric data with ANOVA output**

**Table S2. Measurements of head width across the eyes (µm) and forewing length (in units of head width) in six populations of *Leuctra hippopus*.**

|  | **population** | **sex** | **N** | **Minimum** | **Maximum** | **Mean** | **Std. Deviation** |
| --- | --- | --- | --- | --- | --- | --- | --- |
| **head width** | **Rekem** | **female** | **1** | **1062** | **1062** | **1062.0** |  |
| wing length | Rekem | female | 1 | 6.06 | 6.06 | 6.06 |  |
| head width | Rekem | male | 3 | 872 | 982 | 913.7 | 59.65 |
| wing length | Rekem | male | 3 | 5.41 | 5.77 | 5.65 | 0.20 |
| head width | Ringsaker | female | 4 | 832 | 1015 | 911.8 | 76.46 |
| wing length | Ringsaker | female | 4 | 6.37 | 7.72 | 6.84 | 0.63 |
| head width | Ringsaker | male | 1 | 937 | 937 | 937.0 |  |
| wing length | Ringsaker | male | 1 | 6.05 | 6.05 | 6.05 |  |
| head width | Folldal | female | 4 | 846 | 912 | 866.5 | 30.96 |
| wing length | Folldal | female | 4 | 6.83 | 7.12 | 6.98 | 0.12 |
| head width | Folldal | male | 4 | 731 | 799 | 760.0 | 28.35 |
| wing length | Folldal | male | 4 | 6.07 | 6.89 | 6.40 | 0.36 |

74.08

| head width | Femundsenden | female | 3 | 854 | 994 | 938.0 |
| --- | --- | --- | --- | --- | --- | --- |
| wing length | Femundsenden | female | 3 | 6.18 | 6.33 | 6.24 |
| head width | Femundsenden | male | 2 | 812 | 845 | 828.5 |
| wing length | Femundsenden | male | 2 | 6.15 | 6.23 | 6.19 |
| head width | Isterfoss | female | 16 | 1006 | 1131 | 1072.4 |
| wing length | Isterfoss | female | 16 | 4.89 | 5.49 | 5.19 |
| head width | Isterfoss | male | 10 | 891 | 1053 | 957.5 |
| 0wing length | Isterfoss | male | 10 | 4.25 | 5.1 | 4.77 |
| head width | Vardø | female | 10 | 739 | 958 | 859.0 |

0.08

23.33

0.06

35.05

0.16

50.88

0.23

69.06

| wing length | Vardø | female | 10 | 5.84 | 7.57 | 6.83 | 0.46 |
| --- | --- | --- | --- | --- | --- | --- | --- |
| head width Vardø male 10 700 910 801.3 64.22 | | | | | | | |
| wing length Vardø male 10 6.06 6.92 6.53 0.32 | | | | | | | |

**SPSS output One-way ANOVA with Tukey's post-hoc test**

# Oneway

## [DataSet1] M:\morphometry\spss\hippopus_head_wing2,.sav

**sex = female**

### ANOVAa

|  | | Sum of Squares | df | Mean Square | F | Sig. |
| --- | --- | --- | --- | --- | --- | --- |
| head_width_um | Between Groups | 339837,198 | 2 | 169918,599 | 56,610 | ,000 |
|  | Within Groups | 102052,477 | 34 | 3001,543 |  |  |
|  | Total | 441889,676 | 36 |  |  |  |
| wing_hw | Between Groups | 22,776 | 2 | 11,388 | 85,145 | ,000 |
|  | Within Groups | 4,547 | 34 | ,134 |  |  |
|  | Total | 27,323 | 36 |  |  |  |

a. sex = female

# Post Hoc Tests

### Multiple Comparisonsa

Tukey HSD

| Dependent Variable | (I) loc2 | (J) loc2 | Mean Difference (I-J) | Std. Error | Sig. |
| --- | --- | --- | --- | --- | --- |
| head_width_um | Other Hedmark | Isterfoss | -169,92045* | 21,45843 | ,000 |
|  |  | Vardø | 43,45455 | 23,93788 | ,180 |
|  | Isterfoss | Other Hedmark | 169,92045* | 21,45843 | ,000 |
|  |  | Vardø | 213,37500* | 22,08508 | ,000 |
|  | Vardø | Other Hedmark | -43,45455 | 23,93788 | ,180 |
|  |  | Isterfoss | -213,37500* | 22,08508 | ,000 |
| wing_hw | Other Hedmark | Isterfoss | 1,53435* | ,14324 | ,000 |
|  |  | Vardø | -,09977 | ,15979 | ,808 |
|  | Isterfoss | Other Hedmark | -1,53435* | ,14324 | ,000 |
|  |  | Vardø | -1,63413* | ,14742 | ,000 |
|  | Vardø | Other Hedmark | ,09977 | ,15979 | ,808 |
|  |  | Isterfoss | 1,63413* | ,14742 | ,000 |

### Multiple Comparisonsa

Tukey HSD

| Dependent Variable | (I) loc2 | (J) loc2 | 95% Confidence Interval | |
| --- | --- | --- | --- | --- |
|  |  |  | Lower Bound | Upper Bound |
| head_width_um | Other Hedmark | Isterfoss | -222,5029 | -117,3380 |
|  |  | Vardø | -15,2037 | 102,1128 |
|  | Isterfoss | Other Hedmark | 117,3380 | 222,5029 |
|  |  | Vardø | 159,2569 | 267,4931 |
|  | Vardø | Other Hedmark | -102,1128 | 15,2037 |
|  |  | Isterfoss | -267,4931 | -159,2569 |
| wing_hw | Other Hedmark | Isterfoss | 1,1834 | 1,8854 |
|  |  | Vardø | -,4913 | ,2918 |
|  | Isterfoss | Other Hedmark | -1,8854 | -1,1834 |
|  |  | Vardø | -1,9954 | -1,2729 |
|  | Vardø | Other Hedmark | -,2918 | ,4913 |
|  |  | Isterfoss | 1,2729 | 1,9954 |

*. The mean difference is significant at the 0.05 level.

a. sex = female

# Homogeneous Subsets

Tukey HSDb,c

### head_width_uma

| loc2 | N | Subset for alpha = 0.05 | |
| --- | --- | --- | --- |
|  |  | 1 | 2 |
| Vardø | 10 | 859,0000 | 1072,3750 |
| Other Hedmark | 11 | 902,4545 |  |
| Isterfoss | 16 |  |  |
| Sig. |  | ,146 | 1,000 |

Means for groups in homogeneous subsets are displayed.

1. sex = female
2. Uses Harmonic Mean Sample Size = 11,839.
3. The group sizes are unequal. The harmonic mean of the group sizes is used. Type I error levels are not guaranteed.

Tukey HSDb,c

**wing_hwa**

| loc2 | N | Subset for alpha = 0.05 | |
| --- | --- | --- | --- |
|  |  | 1 | 2 |
| Isterfoss | 16 | 5,1914 | 6,7257 |
| Other Hedmark | 11 |  |  |
| Vardø | 10 |  | 6,8255 |
| Sig. |  | 1,000 | ,786 |

Means for groups in homogeneous subsets are displayed.

1. sex = female
2. Uses Harmonic Mean Sample Size = 11,839.
3. The group sizes are unequal. The harmonic mean of the group sizes is used. Type I error levels are not guaranteed.

# sex = male

### ANOVAa

|  | | Sum of Squares | df | Mean Square | F | Sig. |
| --- | --- | --- | --- | --- | --- | --- |
| head_width_um | Between Groups | 150804,395 | 2 | 75402,197 | 20,109 | ,000 |
|  | Within Groups | 89993,457 | 24 | 3749,727 |  |  |
|  | Total | 240797,852 | 26 |  |  |  |
| wing_hw | Between Groups | 17,739 | 2 | 8,870 | 108,750 | ,000 |
|  | Within Groups | 1,957 | 24 | ,082 |  |  |
|  | Total | 19,696 | 26 |  |  |  |

a. sex = male

# Post Hoc Tests

### Multiple Comparisonsa

Tukey HSD

| Dependent Variable | (I) loc2 | (J) loc2 | Mean Difference (I-J) | Std. Error | Sig. |
| --- | --- | --- | --- | --- | --- |
| head_width_um | Other Hedmark | Isterfoss | -152,64286* | 30,17695 | ,000 |
|  |  | Vardø | 3,55714 | 30,17695 | ,992 |
|  | Isterfoss | Other Hedmark | 152,64286* | 30,17695 | ,000 |
|  |  | Vardø | 156,20000* | 27,38513 | ,000 |
|  | Vardø | Other Hedmark | -3,55714 | 30,17695 | ,992 |
|  |  | Isterfoss | -156,20000* | 27,38513 | ,000 |
| wing_hw | Other Hedmark | Isterfoss | 1,52124* | ,14074 | ,000 |
|  |  | Vardø | -,24706 | ,14074 | ,206 |
|  | Isterfoss | Other Hedmark | -1,52124* | ,14074 | ,000 |
|  |  | Vardø | -1,76830* | ,12772 | ,000 |
|  | Vardø | Other Hedmark | ,24706 | ,14074 | ,206 |
|  |  | Isterfoss | 1,76830* | ,12772 | ,000 |

### Multiple Comparisonsa

Tukey HSD

| Dependent Variable | (I) loc2 | (J) loc2 | 95% Confidence Interval | |
| --- | --- | --- | --- | --- |
|  |  |  | Lower Bound | Upper Bound |
| head_width_um | Other Hedmark | Isterfoss | -228,0034 | -77,2824 |
|  |  | Vardø | -71,8034 | 78,9176 |
|  | Isterfoss | Other Hedmark | 77,2824 | 228,0034 |
|  |  | Vardø | 87,8115 | 224,5885 |
|  | Vardø | Other Hedmark | -78,9176 | 71,8034 |
|  |  | Isterfoss | -224,5885 | -87,8115 |
| wing_hw | Other Hedmark | Isterfoss | 1,1698 | 1,8727 |
|  |  | Vardø | -,5985 | ,1044 |
|  | Isterfoss | Other Hedmark | -1,8727 | -1,1698 |
|  |  | Vardø | -2,0872 | -1,4494 |
|  | Vardø | Other Hedmark | -,1044 | ,5985 |
|  |  | Isterfoss | 1,4494 | 2,0872 |

*. The mean difference is significant at the 0.05 level.

a. sex = male

# Homogeneous Subsets

Tukey HSDb,c

### head_width_uma

| loc2 | N | Subset for alpha = 0.05 | |
| --- | --- | --- | --- |
|  |  | 1 | 2 |
| Vardø | 10 | 801,3000 | 957,5000 |
| Other Hedmark | 7 | 804,8571 |  |
| Isterfoss | 10 |  |  |
| Sig. |  | ,992 | 1,000 |

Means for groups in homogeneous subsets are displayed.

1. sex = male
2. Uses Harmonic Mean Sample Size = 8,750.
3. The group sizes are unequal. The harmonic mean of the group sizes is used. Type I error levels are not guaranteed.

Tukey HSDb,c

**wing_hwa**

| loc2 | N | Subset for alpha = 0.05 | |
| --- | --- | --- | --- |
|  |  | 1 | 2 |
| Isterfoss | 10 | 4,7659 | 6,2871 |
| Other Hedmark | 7 |  |  |
| Vardø | 10 |  | 6,5342 |
| Sig. |  | 1,000 | ,188 |

Means for groups in homogeneous subsets are displayed.

1. sex = male
2. Uses Harmonic Mean Sample Size = 8,750.
3. The group sizes are unequal. The harmonic mean of the group sizes is used. Type I error levels are not guaranteed.
