# Supplementary material for: Ecological speciation by temporal isolation in a population of the stonefly Leuctra hippopus (Plecoptera, Leuctridae)
Source: Ecol Evol. 2017 Feb 10;7(5):1635–49. doi: 10.1002/ece3.2638 (PMC5330929; doi:10.1002/ece3.2638)
Supplement: Supplementary file 6 [file ECE3-7-1635-s006.doc]

# Appendix S2 Notes on taxonomy and phylogeography

## Monophyly of Leuctra hippopus

Lillehammer (1974) and Ørmen (1991) implicitly casted doubt about the taxonomic validity of *L. hippopoides* and *L. elisabethae* when they noted that their morphological characteristics show overlap with Norwegian variants of *L. hippopus*. Moreover, specimens which are morphologically intermediate between *L. hippopus* and *L. hippopoides* have been found in Greece (Berthélemy 1970; Zwick 1978). Our mitochondrial gene tree based on COI supports the monophyly of *L. hippopus* relative to these close relatives as well as *L. andalusiaca* and *L. pseudohippopus*. The nuclear 28S data matrix included *L. hippopus*, *L. elisabethae* and *L. andalusiaca*, which each have slightly distinct alleles. However, it is plausible that some of the taxa closely related to *L. hippopus* but with a narrow distribution range originated as ecologically isolated populations of the latter (Ørmen 1991). A case in point may be *L. hippopoides* in the Balkans. This species is restricted to headwaters with constant low temperature, whereas *L. hippopus* occupies lower parts of the same streams where temperature rises considerably during the day (Kaćanski 1971). A further sampling of *L. hippopus* mitochondrial haplotypes in the Balkan, Anatolia and the Caucasus may show this species to be paraphyletic with respect to *L. hippopoides*.

## Taxonomic status of the Isterfoss population

The question arises whether the Isterfoss population of *L. hippopus* should have species status. No evidence for admixture between Isterfoss and adjacent populations has been found, which indicates effective isolating barriers. Because the Isterfoss population is adapted to an unusual local habitat, any individuals of mixed origin will likely suffer low viability as they are less adapted to the habitat of either parent. This is a likely postzygotic isolating barrier. In spite of these arguments, we are inclined not to consider this population as a distinct species because the morphological differences are quantitative, and with the current knowledge the Isterfoss population cannot be readily diagnosed by morphological or even molecular characters. Instead we emphasise temporal isolation as an ongoing speciation process.

## Phylogeography of L. hippopus

The phylogeny based on COI suggests that *L. hippopus* dispersed northward from at least three glacial refugia, namely the Iberian and Apennine Peninsulas and either the Balkan or the Caucasus, reminiscent of the dispersal routes inferred for the European brown bear, *Ursus arctos* (Hewitt 2000). At this point is it is not clear whether *L. hippopus* dispersed to northern Scandinavia from the Balkan Peninsula or rather from the Caucasus like the brown bear. This will likely become clear when further haplotypes become available from either the Caucasus or the eastern European region spanning from Hungary to Finland. The mitochondrial phylogeny need not be representative of the organism as a whole (Boumans & Tierno de Figueroa 2016). In the case of *L. hippopus*, AFLP and RAD sequences also show a major divide between Vardø and the western European populations. However, it is possible that northwestern European populations are a mixture of Iberian and eastern lineages. This might explain why the Structure analysis of 529 SNPs scored in all six collecting sites associates the three Belgian specimens with Vardø when the number of clusters was set as 2 (main text Error: Reference source not foundb).

## References

Berthélemy C (1970) Plécoptères de Grèce Centrale et d’Eubée. *Biologia Gallo-Hellenica* **3**, 23-56.

Boumans L, Tierno de Figueroa JM (2016) Introgression and species demarcation in western European *Leuctra fusca* (L.,1758) and *L. digitata* Kempny, 1899 (Plecoptera: Leuctridae). *Aquatic Insects*.

Hewitt G (2000) The genetic legacy of the Quaternary ice ages. *Nature* **405**, 907-913.

Kaćanski D (1971) Idioekološke razlike vrste Leuctra hippopus Kempny i Leuctra hippopoides Kaćanski et Zwick (Plecoptera) [Idioecological differences between species Leuctra hippopus Kempny and Leuctra hippopoides Kaćanski and Zwick (Plecoptera), in Serbo-Croatian]. *Ekologija* **6**, 361-374.

Lillehammer A (1974) Norwegian stoneflies. I. Analysis of the variations in morphological and structural characters used in taxonomy. *Norsk Entomologisk Tidsskrift* **21**, 59-107.

Zwick P (1978) Steinfliegen (Insecta, Plecoptera) aus Griechenland und benachbarten Ländern—2. Teil. *Mitteilungen der Schweizerischen Entomologischen Gesellschaft* **51**, 213-239.

Ørmen T (1991) *Leuctra hippopus Kempny, 1899 (Plecoptera: Leuctridae): studies on a species problem, with notes on related species*, University of Oslo.
