# Supplementary material for: Ecological speciation by temporal isolation in a population of the stonefly Leuctra hippopus (Plecoptera, Leuctridae)
Source: Ecol Evol. 2017 Feb 10;7(5):1635–49. doi: 10.1002/ece3.2638 (PMC5330929; doi:10.1002/ece3.2638)
Supplement: Supplementary file 7 [file ECE3-7-1635-s007.doc]

# S3 Extended AFLP and RAD sequence methods

## Phylogenetic analysis of COI sequences

The distance measure for the neighbour joining method in Paup* was set as ‘dist=GTR rates=gamma shape=1.9366 Pinvar=0.6754’, based on the evolution model selected under the Akaike information criterion with MrModeltest 2.2 (Nylander 2004). We carried out heuristic searches under the optimality criteria distance and parsimony with tree bisection-reconnection branch swapping and 100 random addition sequence replicates. Bootstrapping (2000 replicates) was performed to obtain support values for branches. For Bayesian inference, the COI data were divided in a partition for the first and second codon position, and one for the third position. Based on the Akaike information criterion, the evolution model GTR+I was selected for the former, and GTR+G for the latter partition. We ran two independent analyses, each consisting of four Markov chains that ran for 40 × 106 generations and were sampled every 1000 generations, with default priors and partition-specific substitution rates (setting prset ratepr=variable). After discarding the first 10 million generations, remaining trees from both analyses were combined and a 50% majority rule consensus tree was calculated. MrBayes output files and Tracer v1.5.0 (Drummond & Rambaut 2007) were used to inspect trace plots and convergence diagnostics (average standard deviation of split frequencies < 0.01, effective sample size > 200) in order to ensure that the Markov chains had reached statistical stationarity and converged on the parameter estimates and tree topology after the burn-in phase, which was set at 25%. Calculations in MrModeltest, Paup* and MrBayes were performed at the Lifeportal computer facility (www.lifeportal.uio.no) at the University of Oslo, Norway.

## AFLP fingerprinting protocol

We determined DNA concentration by means of gel electrophoresis as well as ﬂuorimetry using the Picodrop® Microliter UV/Vis Spectrophotometer. For restriction-ligation, 5 μl extracted DNA was mixed with 1X T4 buffer (Roche, Mannheim, Germany), 0.05 M NaCl, 0.55 μg bovine serum albumin (BSA), 1 U Mse, 5 U *Eco*RI, 1 U T4 DNA ligase, 0.9 μM *Mse* Adaptor, and 0.9 μM *Eco* Adaptor to a final volume of 10 μl. The adaptors were denatured at 95 °C for 5 min and annealed by slow cooling to room temperature before adding them to the reaction mix. The mixture was incubated for 2-3 h at 37 °C and diluted 10 times with purified water. The 12.5 μl preselective PCR reaction mix contained 1.5 μl diluted restriction-ligation product, 1X Taq buffer, 1.5 mM MgCl2, 0.02 mM of each dNTP, 0.2 μM *Eco*RI-A primer, 0.2 μM *Mse*I-C primer, and 0.37 U AmpliTaq polymerase (Applied Biosystems). The preselective PCR conditions were 2 min at 72 °C followed by 30 cycles of 30 s at 94 °C, 30 s at 56 °C, and 1 min at 72 °C, with a final 10-min extension at 72 °C. The pre-selective amplification product was diluted 17.7 times with purified water. The 12.5 μl selective PCR reaction mix contained 2.5 μl of the diluted pre-selective amplification product, 1X AmpliTaq Gold buffer (Applied Biosystems), 2.5 mM MgCl2, 0.2 mM of each dNTP, 0.08 μM *Eco*RI-AXX fluorescence labelled primer, 0.2 μM *Mse*I-CXX primer, 0.5 U AmpliTag Gold polymerase (Applied Biosystems), and 0.1 μg BSA.

We tested ten primer combinations with three selective bases for both *Eco*RI and *Mse*I in sixteen individuals representing the five sampling sites. In the primer test phase, seven individuals were run twice in order to test reproducibility of the markers. We selected the three primer combinations 6FAM-*Eco*RI-ACA + *Mse*I-CAT, VIC-*Eco*RI-ACG + *Mse*I-CAA and NED-*Eco*RI-AGA + *Mse*I-CTG, as these had the best separated and polymorphic fragments while scoring best in the reproducibility test. Both pre-selective and selective PCRs were run on a GeneAmp PCR system 9700 thermocycler. For the selective PCR, ramp speed was set to 90%.

We loaded the three chosen primer combinations together and analysed them on an ABI 3100 DNA Sequencer using 3.5 μl of selective PCR products (from a mix of 2.0 μl 6-FAM, 2.0 μl VIC, 3.0 μl NED and 14 μl mqH_2_O), 0.3 μl GeneScan ROX 500 (Applied Biosystems) and 11.7 μl HiDi (formamide) per run. The raw data were aligned with the ROX 500 size standard and visualized in Genographer version 2.1.4 and in GeneMapper® Software analysis software (Applied Biosystems 2004).

Eight of the 56 individuals yielded only a small number of amplicons in one or more of the three PCR reactions. These were excluded, leaving 48 individuals and thirteen duplicates (27%). Duplicates served to calculate error rates, defined as “the ratio between observed number of phenotypic differences and total number of phenotypic comparisons” (Bon*in et a*l. 2004: 3262) with the formula (total No. of mismatches)*(No. of repeated samples)^-1^*(No. of markers)^-1^.

As scoring by means of visual inspection introduces a high degree of subjectivity (Bon*in et a*l. 2004), we chose an automated approach for the scoring of markers as either absent of present. We output unedited peak heights in the size range from 50 to 500 bp from GeneMapper and analysed these with the R program AFLPScore version 1.4 (Whitlo*ck et a*l. 2008), which normalises signal intensities of individual samples prior to scoring a marker as present or absent. No pre-set value for the normalisation was provided, as all data were analysed in a single table. The program’s Filtering function, which reduces noise from spurious peaks below the phenotype-calling threshold, was applied.

For each primer pair, markers were scored as present or absent in two iterations: First, the mismatch error rate was assessed for a range of thresholds, starting with values of zero to twice the Grand mean normalized peak height (in relative fluorescence units RFU) for the locus selecting threshold, and 0-100% the Grand mean normalized peak height for the relative phenotype threshold. The optimal threshold settings were identified by the mismatch percentage and the number of loci scored. With the optimal settings () a genotypes table was output. A calculation sheet was used to reduce the total error rate to a value <2.5%. To this end, markers with inconsistent signal were deleted. The list of retained markers was used to perform a second step of threshold selection and phenotype scoring in AFLPScore. These rescored phenotypes were again filtered until the error rate was below 2%. Subsequently, we removed markers present in only a single sample, which led to a final error rate of 2.8–3.2% for the three reactions ( S3.1).

Among the replicated samples, we retained those that had most positive markers, since a false positive marker is less likely than a false negative. The dataset was checked for the occurrence of (actual or artificial) linked markers resulting from minimal size difference, visible as double peaks in GeneMapper. Potential cases were identified by creating a transposed version of the data matrix and clustering the markers in a neighbour joining tree using matching as distance coefficient in the program Paup*, following Westergaa*rd et a*l. (2010). Closely clustered markers with minimal fragment length difference amplified by the same primer pair are potential artefacts, but no such linked markers were identified. The resulting dataset contained 109 markers. Each of the 48 remaining samples had 19.7 ± 4.5 markers scored as present (range 9–28).

Table S3.1. Scoring of AFLP markers.

|  | Initial automated scoring | | | | Set most replicable loci | | Rescoring subset 1 | | Final subset | |
| --- | --- | --- | --- | --- | --- | --- | --- | --- | --- | --- |
| Selective primer pair | locus selection threshold 1 (relative fluorescence unit) | phenotype selection threshold 1  % of mean rfu for marker | initial nr. polymorphic of markers (N duplicates) | initial error rate* | subset 1 (N duplicates) | error rate subset 1 | locus selection threshold 2 (relative fluorescence unit) | phenotype selection threshold 2  % of mean rfu for marker | final nr of markers with >1 band | final error rate (N duplicates) |
| FAM | 35 | 35% | 198 (13) | 14.0% | 68 (13) | 1.8% | 32.5 | 30% | 44 | 2.8% (13) |
| VIC | 30 | 40% | 199 (13) | 16.2% | 52 (13) | 2.0% | 25 | 37.5 | 33 | 3.2% (13) |
| NED | 20 | 17% | 124(13) | 11.4% | 53 (13) | 2.4% | 20 | 17.5% | 32 | 3.1% (13) |
| sum |  |  |  |  |  |  |  |  | 109 |  |

## RAD sequence preparation

We followed a modified version of the protocol in Etter et al. (2011). The adaptor and PCR primer conditions are listed in section two of this Supplementary Materials. The library protocol can be divided into the following steps:

1) DNA extraction: The insect’s abdomen was removed to avoid contamination with intestinal flora and fauna. DNA was extracted from the remainder of the insect with DNeasy blood and tissue kit (Qiagen), and the protocol was followed with some modifications. The samples were left in a heating cabinet at 56°C overnight with proteinase K. Then 20µL of RNase was added and incubated at room temperature for two minutes, before continuing with the extraction. After the DNA was bound to the spin columns, the samples were dried at 50°C for approximately 5 minutes to remove all extra ethanol. Then 60µL EB-buffer was added in the elution step and left at 60°C for 10 minutes, before spinning the DNA down.

2) Digestion with SdfI-HF restriction enzyme (New England Biolabs): The samples were incubated with a master mix containing CutSmart Buffer (1X), SdfI-HF (200U/mL), dH2O and DNA (50-500ng) for 1.5 hours at 37°C, and subsequently at 65°C for 20 minutes to inactivate the enzyme.

3) P1 adapter ligation: Adapter ligation was performed in the same tubes as digestion reaction using 9mM P1 adaptor, CutSmart buffer (0,2X), rATP (1mM), T4 DNA ligase (16666U/mL) and dH2O. This mixture was incubated at room temperature for 30 minutes, before the T4 DNA ligase was inactivated at 65°C for 20 minutes. See table S1 for individual samples and their adaptor.

4) Multiplexing and shearing: All samples were pooled and divided into two sonication tubes; one of these was randomly sheared (Biorupter) to an average size of 500bp (the other was preserved as back-up at -20°C). The sample was run seven cycles on a 30 seconds on and 30 seconds off interval. The sonicated samples were cleaned with MinElute columns and a Qiagen PCR purification kit following the manufacturer’s protocol.

5) Size selection and gel extraction: The purified sonicated product was run on an agarose gel (1.25%) at 100V for 50 minutes. The band between 250-500bp was cut and cleaned with MinElute gel purification kit (Qiagen) following the manufacturer’s protocol. The end product was 20uL EB buffer eluted into a tube containing 1.5uL (10x) Blunting Buffer (Qiagen).

6) Repair fragments after sonication: The Quick Blunting protocol was used with T4 DNA Polymerase and T4 Polymerase Kinase to convert 5’ and 3’ overhangs into phosphorylated blunt ends, and incubated at room temperature for 30 minutes. To prepare the DNA fragments for ligation to P2 adapters, an A base was added to the 3’ end by using Klenow activity polymerase (New England Biolabs) and incubation at 37°C for 30 minutes.

7) Bead purification and P2 adaptor ligation: The sample was purified with beads to get rid of all fragments below 200bp. Then a P2 adaptor was added to the sample. This adaptor contains a Y structure that only binds the P2 primer to the DNA fragment if the P1 adaptor has been ligated to the product, and serves to distinguish target from non-target fragments. The P2 adaptor was ligated with T4 DNA ligase, P2 adaptor and rATP. The mixture was incubated at room temperature for 30 minutes. Then the product was cleaned once more with beads.

8) RAD tag amplification: Amplification reactions were performed with Phusion High Fidelity Master mix (New England Biolabs), Solexa primer mix and the RAD library template, under the following conditions: 98°C for 30 seconds; 21 cycles with: 98°C for 10 seconds, 65°C for 30 seconds, 72°C for 30 seconds and a final step with 72°C for 5 minutes. First, we ran one test using 44 µL of the RAD library as template and the master mix and conditions as described. The amplicons were then run on an agarose gel (1%) for 30 minutes at 90 V to test if the reaction had worked. Then this step was repeated with the remaining RAD library template. The final volume was split into eight aliquots to avoid drift before running the product on the PCR machine. Then final bead purification was run before the RAD library was sent to the Norwegian Sequencing Center at Ullevål University Hospital, University of Oslo, Norway.

A table with the barcode sequences used to identify the individual stonefly specimens is included with the RAD tag reads in the digital repository.

**References**

Bonin A, Bellemain E, Bronken Eidesen P*, et al.* (2004) How to track and assess genotyping errors in population genetics studies. *Molecular Ecology* **13**, 3261-3273.

Drummond A, Rambaut A (2007) BEAST: Bayesian evolutionary analysis by sampling trees. *BMC evolutionary biology* **7**, 214.

Etter PD, Preston JL, Bassham S, Cresko WA, Johnson EA (2011) Local De Novo Assembly of RAD Paired-End Contigs Using Short Sequencing Reads. *Plos One* **6**, e18561.

Nylander JAA (2004) MrModeltest v2. Program distributed by the author, Evolutionary Biology Centre, Uppsala University.

Westergaard KB, Jørgensen MH, Gabrielsen TM, Alsos IG, Brochmann C (2010) The extreme Beringian/Atlantic disjunction in Saxifraga rivularis (Saxifragaceae) has formed at least twice. *Journal of Biogeography* **37**, 1262-1276.

Whitlock R, Hipperson H, Mannarelli M, Butlin R, Burke T (2008) An objective, rapid and reproducible method for scoring AFLP peak‐height data that minimizes genotyping error. *Molecular Ecology Resources* **8**, 725-735.
